# Recent advances in magnetic nanocatalysts for synthesis of imidazo[1,2-*a*]pyridine frameworks

**DOI:** 10.1039/d5ra08924g

**Published:** 2026-01-02

**Authors:** Mohamed Abu Shuheil, G. Padma Priya, Amaal Mohammed Ali, Y. Sasikumar, Ahmed Aldulaimi, Renu Sharma, Abhayveer Singh, Radwan Ali, Mosstafa Kazemi

**Affiliations:** a Faculty of Allied Medical Sciences, Hourani Center for Applied Scientific Research, Al-Ahliyya Amman University Amman Jordan m.abushuhel@ammanu.edu.jo; b Department of Chemistry and Biochemistry, School of Sciences, JAIN (Deemed to Be University) Bangalore Karnataka India g.padmapriya@jainuniversity.ac.in; c Department of Optics Techniques, Health and Medical Techniques College, Alnoor University Mosul Iraq amaal.mohammed@alnoor.edu.iq; d Department of Chemistry, Sathyabama Institute of Science and Technology Chennai Tamil Nadu India sasikumar.chemistry@sathyabama.ac.in; e Faculty of Pharmacy, Al-Zahrawi University Karbala Iraq Ahmedaldulaimi1@gmai.com; f Department of Chemistry, University Institute of Sciences, Chandigarh University Mohali Punjab India drrenusharma01@outlook.com; g Faculty of Science, Gokul Global University Sidhpur Gujarat India abhayveer_singh@outlook.com; h Al-Qadisiyah University, College of Dentistry, Department of Basic Sciences Al-Qadisiyah Iraq radwan.ali@qu.edu.iq; i Young Researchers and Elite Club, Tehran Branch, Islamic Azad University Tehran Iran mosstafakazemi@gmail.com

## Abstract

Magnetic nanocatalysts (MNCs) have emerged as powerful and sustainable catalytic platforms for the synthesis of imidazo[1,2-*a*]pyridine derivatives, an important class of nitrogen-fused heterocycles with broad applications in medicinal chemistry, materials science, and agrochemistry. Their nanoscale dimensions, high surface area, and chemically tailorable surfaces provide enhanced catalytic activity, while their magnetic cores enable rapid and efficient recovery using an external magnet, eliminating the need for energy-intensive purification steps. Recent advances demonstrate that Fe_3_O_4_, CoFe_2_O_4_, γ-Fe_2_O_3_, and related magnetic nanomaterials—either alone or functionalized with metals, ligands, ionic liquids, or polymeric shells—can effectively promote classical condensations, Groebke–Blackburn–Bienaymé multicomponent reactions, Aldehyde–Amine–Alkyne coupling (A^3^-couplings), and oxidative cyclizations leading to diverse imidazo[1,2-*a*]pyridines under mild, green conditions. These catalysts often exhibit excellent stability, minimal metal leaching, and reusability across multiple cycles without significant loss of activity. Mechanistically, magnetic nanocatalysts facilitate carbonyl activation, imine formation, alkyne or isocyanide activation, and intramolecular cyclization, enabling high yields and broad substrate tolerance. Compared with conventional catalysts, they offer superior efficiency, selectivity, and environmental compatibility. This review summarizes key developments in magnetically recoverable catalytic systems for imidazo[1,2-*a*]pyridine synthesis and highlights their growing significance in advancing sustainable heterocyclic chemistry.

## Abstract 2

Magnetic nanocatalysts (MNCs) represent sustainable platforms for synthesizing imidazo[1,2-*a*]pyridine derivatives, key nitrogen-fused heterocycles with wide applications in medicinal chemistry, materials science, and agrochemistry.

## Graphical abstract

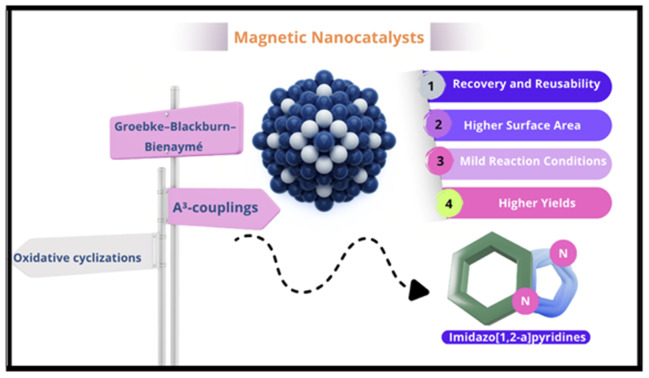

## 1. Introduction

Catalysts are fundamental to modern chemistry because they enable and accelerate chemical reactions that might otherwise proceed extremely slowly or not at all under normal conditions.^1,2^ Catalysts serve as facilitators in the intricate dance of chemical reactions, introducing an alternative pathway that significantly lowers the activation energy required.^3,4^ By doing so, they diminish the energy barrier that reactant molecules must surmount to evolve into products. This remarkable capability accelerates the reaction process, allowing it to unfold swiftly—even under milder conditions of temperature and pressure.^5,6^ Such efficiency is invaluable, making catalysts indispensable in both laboratory experiments and large-scale industrial applications, where speed and reduced energy consumption are paramount.^7^ Catalysts function by adsorbing reactant molecules onto their active sites, facilitating bond breaking and formation through intermediate species, and subsequently releasing the final products without undergoing permanent chemical change.^8^ Because they remain unconsumed after the reaction, a small quantity of catalyst can convert a large amount of reactants, making the process both economical and efficient.^9,10^ Beyond simply increasing reaction rates, catalysts also profoundly influence reaction selectivity and yield. In industrial applications, catalytic systems are tailored to produce specific products while minimizing undesired by-products, thereby improving overall process efficiency and reducing waste.^11^ Catalysts are central to key technologies such as hydrogenation, oxidation, polymerization, and environmental control reactions like catalytic converters in automobiles. As a result, catalysts serve as the dynamic agents of chemical innovation, transforming raw materials into groundbreaking products while simultaneously championing the advancement of sustainable industrial practices.^12^ They are instrumental in forging global energy solutions, guiding our journey toward a greener future with their vital contributions.

### 1.1. Heterogeneous and homogeneous catalysts

Heterogeneous catalysts are dynamic agents that exist in a different phase from the reactants, typically as solid forms interacting with liquid or gaseous substances.^13^ This remarkable interplay allows for enhanced reaction rates and selectivity in various industrial processes, showcasing the intricate dance between different states of matter.^14,15^ These catalysts function by providing active surface sites where reactant molecules can adsorb, react, and then desorb as products.^16^ The catalytic process typically involves several steps—adsorption of reactants, surface reaction, and desorption of products—all occurring at the catalyst's surface.^17,18^ Materials such as metals (*e.g.*, palladium, platinum, nickel), metal oxides (*e.g.*, TiO_2_, Al_2_O_3_), and supported nanocomposites (*e.g.*, metal nanoparticles dispersed on carbon or silica) are widely used as heterogeneous catalysts.^19^ The physical and chemical properties of these materials, including surface area, pore structure, and dispersion of active sites, directly influence their catalytic performance.^20^

Heterogeneous catalysts, existing in a distinct phase separate from the reactants, offer the significant advantage of easy separation from the reaction mixture through methods such as filtration or magnetic separation.^20,21^ This characteristic not only facilitates their recovery but also enhances their recyclability, ultimately minimizing waste and lowering operating costs.^22^ Moreover, these catalysts exhibit remarkable stability when subjected to various reaction conditions, allowing them to be utilized repeatedly without a notable decline in their catalytic activity.^23^ Their enduring efficiency makes them a valuable asset in industrial processes, promoting sustainability and cost-effectiveness. Moreover, heterogeneous systems are generally better suited to large-scale, continuous-flow industrial processes due to their robustness and ease of handling.^24,25^ In contrast, homogeneous catalysts often require complex and energy-intensive separation and purification steps. Although homogeneous catalysts sometimes offer higher selectivity due to their molecular-level uniformity, the recent development of nanostructured heterogeneous catalysts has significantly bridged this gap by providing high activity, tunable selectivity, and excellent reusability, making them key components in modern green and sustainable catalytic systems.^26,27^

Nanomaterials are advanced catalytic materials composed of two or more distinct phases, at least one of which possesses nanometer-scale dimensions, designed to integrate the structural and functional benefits of each component.^28–31^ In catalytic systems, nanocomposites often combine metal or metal oxide nanoparticles with supporting materials such as carbon nanotubes, graphene, silica, or polymers to create a hybrid structure with enhanced activity, stability, and selectivity.^32^ Their high surface area-to-volume ratio provides numerous accessible active sites, while the interaction between components facilitates efficient charge transfer and molecular adsorption during reactions.^33^ By preventing nanoparticle agglomeration and promoting uniform dispersion, nanocomposite catalysts exhibit superior durability and recyclability compared with conventional catalysts, making them highly valuable in heterogeneous catalysis, green synthesis, and environmental remediation.^34^

### 1.2. Magnetic nanocatalysts

Magnetic nanocatalysts (MNCs) represent a rapidly developing field in modern catalysis, offering an efficient combination of nanotechnology and green chemistry.^29,35^ These materials typically consist of a magnetic core—such as Fe_3_O_4_, γ-Fe_2_O_3_, CoFe_2_O_4_, or NiFe_2_O_4_—coated or functionalized with catalytically active species such as metals (Pd, Pt, Ni, Cu) or metal complexes.^36,37^ Magnetic nanocatalysts can be quickly separated from a reaction mixture because their magnetic cores respond to an external magnetic field.^34,38^ Their nanoscale size provides a large surface area, which boosts catalytic activity and efficiency. Together, these features combine the benefits of both homogeneous and heterogeneous catalysts, making them highly suitable for sustainable, large-scale processes.^39,40^

#### 1.2.1. Advantages of magnetic catalysts

##### 1.2.1.1. High surface area and enhanced catalytic activity

The nanoscale dimensions of magnetic nanoparticles provide an enormous surface area-to-volume ratio, ensuring a high density of active sites accessible to reactant molecules.^41,42^ When catalytic metals or complexes are immobilized on magnetic supports, they become highly dispersed, minimizing aggregation and maximizing efficiency.^43^ This improved surface exposure facilitates rapid mass transfer and electron mobility, resulting in faster reaction rates and lower activation energies.^44^ For example, Fe_3_O_4_-supported Pd and Cu nanocatalysts have shown remarkable activity in C–C coupling reactions, oxidations, and reductions, outperforming bulk catalysts due to their superior atomic utilization and interfacial synergy.^45,46^

##### 1.2.1.2. Easy magnetic separation and recovery

One of the most outstanding features of magnetic nanocatalysts is their magnetic recoverability. In contrast to traditional heterogeneous catalysts that require tedious processes like filtration or centrifugation for separation, magnetic catalysts offer a far more elegant solution.^33,47^ These innovative catalysts can be effortlessly extracted from the reaction mixture with the mere sweep of an external magnet, transforming separation into a swift and seamless operation.^48^ This easy and rapid separation eliminates the need for complex purification procedures, reduces solvent use, and minimizes product contamination.^7,49^ The ability to reuse magnetic nanocatalysts supports sustainable catalytic processes by conserving time and resources while still delivering stable performance across multiple reaction cycles.^50^

##### 1.2.1.3. Superior stability and resistance to deactivation

Magnetic nanoparticles are often coated with stabilizing shells or functional layers—such as silica (SiO_2_), alumina (Al_2_O_3_), carbon, or polymers—to prevent agglomeration, oxidation, and leaching.^51^ This protective layer enhances thermal and chemical stability, ensuring that the catalyst remains active under demanding reaction conditions.^52^ In addition, the coating provides anchoring sites for immobilizing catalytic species, preventing their detachment or dissolution during reactions. Consequently, magnetic nanocatalysts demonstrate long operational lifetimes, maintaining structural integrity and catalytic performance even after repeated uses.^53^

##### 1.2.1.4. Reusability and economic benefits

The recyclability of magnetic nanocatalysts offers significant economic and environmental advantages.^54^ Because they are magnetically separable and structurally stable, these catalysts can be reused repeatedly without significant loss of activity.^55,56^ This reduces the cost associated with precious metal catalysts and minimizes waste production. Studies have shown that magnetic nanocatalysts retain over 90% of their original activity even after ten consecutive reaction cycles.^57,58^ Such long-term reusability makes magnetic nanocatalysts highly attractive for industrial-scale applications where both performance and cost-effectiveness are crucial.^59^

##### 1.2.1.5. Tunable surface chemistry and selectivity

A key advantage of magnetic nanocatalysts is their chemically modifiable surface. Functional groups such as –NH_2_, –SH, –COOH, or silane linkers can be grafted onto the surface to tailor its reactivity, polarity, and coordination ability.^60,61^ These surface modifications allow for precise control over catalytic selectivity, enabling specific interactions between catalysts and substrates. For example, amine-functionalized Fe_3_O_4_ nanoparticles can effectively bind metal ions like Pd^2+^ or Cu^2+^, forming stable complexes that exhibit high selectivity in multicomponent and coupling reactions.^62,63^

##### 1.2.1.6. Environmental compatibility and green chemistry relevance

Magnetic nanocatalysts play a pivotal role in promoting green and sustainable chemical processes.^64^ Their ease of separation and recyclability significantly reduce solvent usage, energy input, and waste generation.^65^ Furthermore, many magnetic nanocatalysts operate efficiently under mild reaction conditions—such as lower temperatures and atmospheric pressure—and in eco-friendly solvents such as water, ethanol, or glycerol.^66,67^ These environmentally benign features minimize the ecological footprint of catalytic processes, aligning with the principles of green chemistry, particularly in terms of waste prevention, catalyst reusability, and safer reaction conditions.^68,69^

##### 1.2.1.7. Versatile applications across chemical fields

Magnetic nanocatalysts have demonstrated outstanding versatility across multiple areas of chemistry and materials science.

###### 1.2.1.7.1. Organic synthesis

Efficient catalysts in coupling, hydrogenation, and oxidation reactions.^70^

###### 1.2.1.7.2. Environmental remediation

Degradation of dyes, pesticides, and organic pollutants in wastewater.^71^

###### 1.2.1.7.3. Energy applications

Involvement in electrocatalysis, fuel cells, photocatalysis, and CO_2_ conversion.^72^

###### 1.2.1.7.4. Biomedical uses

Magnetic separation, biosensing, and targeted drug delivery due to biocompatible coatings.^73^

This wide applicability highlights the multifunctional nature of magnetic nanocatalysts and their potential to solve both industrial and environmental challenges through innovative catalytic design.^74^

##### 1.2.1.8. Bridging homogeneous and heterogeneous catalysis

Magnetic nanocatalysts serve as an intermediate platform between homogeneous and heterogeneous catalysis, integrating the strengths of both.^75,76^ They offer the high activity and selectivity typical of homogeneous catalysts while retaining the easy recovery and reusability associated with heterogeneous systems.^77,78^

As a result, magnetic nanocatalysts stand at the forefront of modern catalysis, offering a combination of efficiency, reusability, tunability, and environmental sustainability.^79^ Their magnetic nature enables easy separation and repeated use, and their nanoscale structure delivers strong catalytic activity and selectivity.^80–82^ The ability to operate under mild and eco-friendly conditions makes them essential tools for green and sustainable synthesis, environmental protection, and energy-related applications.^83,84^ As research continues to advance, the integration of magnetic nanostructures with functional ligands, carbon materials, and polymer supports will further enhance their performance and broaden their applicability.^85^ Ultimately, magnetic nanocatalysts represent a crucial step toward next-generation catalytic systems that combine innovation, practicality, and environmental responsibility.^86^

Magnetic nanocatalysts offer a next-generation solution to traditional catalyst systems by uniting high catalytic efficiency with excellent reusability and environmentally friendly operation Table 1. Their design flexibility and easy magnetic recovery make them ideal for green, sustainable synthesis, thereby reducing both economic and ecological burdens associated with traditional catalysis.^87,88^ While conventional catalysts still dominate large-scale processes due to simplicity and existing industrial infrastructure, the continued advancement of nanostructured magnetic materials is rapidly transforming catalytic technology toward more efficient, eco-friendly, and recyclable systems.^89^

**Table 1:** Comparison between magnetic nanocatalysts and conventional catalysts

Feature	Conventional catalysts	Magnetic nanocatalysts
Physical form	Bulk or microscale materials	Nanometer-sized magnetic cores
Surface area	Low to moderate	Very high
Active site accessibility	Limited	Excellent
Catalyst recovery	Filtration or extraction	Simple magnetic separation
Reusability	Limited or difficult	High (multiple cycles)
Stability	Susceptible to sintering/leaching	Stable due to surface coating
Selectivity control	Difficult to modify	Easily tunable by surface modification
Reaction conditions	Often harsh	Mild and green
Environmental impact	High waste generation	Eco-friendly and sustainable
Economic viability	Higher long-term cost	Cost-effective over multiple uses

### 1.3. Imidazo[1,2-*a*]pyridines

**Fig. 1:**
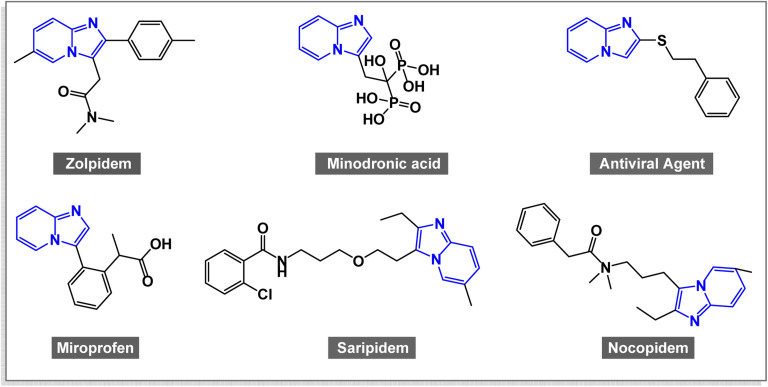
Several examples of bioactive imidazo[1,2-*a*]pyridine derivatives.

Imidazo[1,2-*a*]pyridine is a fused bicyclic heteroaromatic compound composed of an imidazole ring condensed with a pyridine ring.^90^ This structural framework is recognized as a “privileged scaffold” in modern chemistry because of its exceptional electronic delocalization, chemical stability, and pharmacophoric versatility.^90^ It serves as a versatile core for designing functional materials, pharmaceutical agents, agrochemicals, and catalysts.^91^ The ongoing research on imidazo[1,2-*a*]pyridine derivatives highlights their significance in various fields. The combination of robust biological activity and potential for material applications underscores the need for continued investigation into this class of compounds.^92^ Imidazo[1,2-*a*]pyridine derivatives have become highly important in medicinal chemistry because of their wide range of biological activities. Fig. 1 illustrates several representative bioactive compounds from this structural class.^93–95^ Imidazo[1,2-*a*]pyridine derivatives display diverse pharmacological activities, notably anti-inflammatory, anticancer, and antimicrobial effects.^96^ The unique structure of imidazo[1,2-*a*]pyridine allows for the interaction with various biological targets, making them promising candidates for drug development.^97^ Researchers are continuously exploring these derivatives to discover new therapeutic agents that can tackle challenging diseases.

#### 1.3.1. Industrial importance

Imidazo[1,2-*a*]pyridines have gained remarkable attention in industry due to their thermal stability, tunable electronic characteristics, and ease of structural modification.^98^ These properties make them excellent candidates for applications in advanced materials, photochemistry, and industrial catalysis.^99^ The extended conjugation within the heterocyclic framework enables efficient light absorption and emission, which is particularly useful in organic light-emitting diodes (OLEDs), fluorescent dyes, and molecular sensors.^100^ Furthermore, imidazo[1,2-*a*]pyridine units can be incorporated into polymeric matrices or coordination polymers to enhance conductivity and optical performance.

##### 1.3.1.1. Industrial examples

2-Phenylimidazo[1,2-*a*]pyridine derivatives – employed in fluorescent materials and optical sensors for detecting transition metal ions.^101^

Imidazo[1,2-*a*]pyridine-functionalized polymers – utilized in optoelectronic and photovoltaic devices due to their π-conjugated backbone.

Transition-metal complexes (Pd, Cu, Ni) with imidazo[1,2-*a*]pyridine ligands – act as heterogeneous catalysts in oxidation and C–C coupling reactions.^102^

Imidazo[1,2-*a*]pyridines constitute an industrially important N-heterocycle that connects developments in materials science with applications in catalysis.

#### 1.3.2. Biological importance

From a biological standpoint, imidazo[1,2-*a*]pyridines display diverse biochemical interactions arising from their nitrogen-rich, electron-dense framework.^103^ Their heterocyclic nature allows for π–π stacking with nucleic acids, hydrogen bonding with enzyme residues, and coordination with metal ions in biomolecules, resulting in strong biological activity.^104^ They demonstrate a broad spectrum of antimicrobial, antiviral, antioxidant, and antiparasitic properties. Some derivatives show selective inhibition of microbial enzymes and receptors, making them valuable leads in drug discovery.^105^

##### 1.3.2.1. Biological examples

Imidazo[1,2-*a*]pyridine-3-carbaldehyde derivatives – exhibit antibacterial activity against *Staphylococcus aureus* and *E. coli*.^106^

N-substituted imidazo[1,2-*a*]pyridines – display antiviral activity against influenza and herpes simplex viruses.^107^

Halogenated derivatives – show antiparasitic potential against *Plasmodium falciparum* (malaria) and *Leishmania* species.^108^

These properties make the scaffold an attractive molecular motif in biochemical and pharmacological research.

#### 1.3.3. Pharmaceutical importance

Pharmaceutical industries have extensively explored imidazo[1,2-*a*]pyridines as central nervous system (CNS)-active agents, kinase inhibitors, and receptor modulators.^109^ Their fused structure provides rigidity and hydrophobicity, facilitating membrane permeability and metabolic stability. The pharmacophore's adaptability allows substitution at multiple ring positions, enabling fine-tuning of pharmacokinetic and pharmacodynamic profiles.^110^ Several imidazo[1,2-*a*]pyridine derivatives are approved or in clinical development as therapeutic drugs.

##### 1.3.3.1. Key pharmaceutical examples

Zolpidem (Ambien®) – a non-benzodiazepine hypnotic drug acting as a GABA_A receptor modulator for insomnia.^111^

Alpidem – an anxiolytic agent similar to benzodiazepines but with a distinct chemical framework.^112^

Saripidem – a sedative–hypnotic compound with fast CNS activity.^113^

GSK812397 – a CCR5 receptor antagonist with potential in HIV treatment.^113^

Olprinone – a phosphodiesterase-III inhibitor used in acute heart failure management.^114^

Niraparib (MK-4827) – a PARP inhibitor for the treatment of ovarian and breast cancers.^113^

The success of these drugs demonstrates that the imidazo[1,2-*a*]pyridine scaffold is a clinically validated pharmacophore with promising therapeutic diversity.

#### 1.3.4. Agrochemical importance

In the agrochemical field, imidazo[1,2-*a*]pyridines are employed as fungicides, insecticides, herbicides, and plant growth regulators.^115^ Their biological activity stems from their ability to disrupt enzyme systems and inhibit metabolic pathways in pests and pathogens, while maintaining high stability in environmental conditions.^116^ The hydrophobic aromatic core enhances penetration through plant cuticles and insect membranes, making these compounds effective even at low dosages.

##### 1.3.4.1. Representative agrochemical examples

Imidazo[1,2-*a*]pyridine–triazole hybrids – exhibit strong fungicidal activity against *Aspergillus niger* and *Fusarium oxysporum*.^117^

Chlorinated imidazo[1,2-*a*]pyridines – function as herbicidal agents by interfering with photosynthetic electron transport.

Imidazo[1,2-*a*]pyridine–thiazole derivatives act as broad-spectrum insecticides effective against aphids, mosquitoes, and mites.

Imidazo[1,2-*a*]pyridine–pyrimidine analogues – serve as plant growth regulators, enhancing crop productivity.

These findings establish imidazo[1,2-*a*]pyridines as sustainable alternatives to traditional agrochemicals with improved selectivity and biodegradability.

#### 1.3.5. Medicinal importance

Medicinal chemistry recognizes imidazo[1,2-*a*]pyridine as a core structure with multi-target potential.^117^ Owing to its ability to interact with diverse receptors and enzymes, it has been applied to develop anticancer, anti-inflammatory, antitubercular, anticonvulsant, and analgesic drugs.^118^ The molecular rigidity and lipophilic–hydrophilic balance of the scaffold ensure strong receptor binding and optimized pharmacological profiles.

##### 1.3.5.1. Medicinal applications and examples

Anticancer activity: imidazo[1,2-*a*]pyridine-based CHK1 and ATM kinase inhibitors are under investigation for DNA damage response modulation.^113^

Antitubercular drugs: nitroimidazo[1,2-*a*]pyridine analogs of PA-824 exhibit potent action against *Mycobacterium tuberculosis*.^113^

Anti-inflammatory agents: nitro-substituted derivatives inhibit COX-2 and TNF-α pathways.^118^

Anticonvulsant activity: 2-substituted derivatives show GABAergic modulation, reducing seizure frequency.^118^

Antiviral and antibacterial potential includes inhibition of bacterial topoisomerase and viral polymerase enzymes.^119^

Hence, the imidazo[1,2-*a*]pyridine skeleton continues to inspire next-generation therapeutic agents targeting complex diseases.

#### 1.3.6. Chemical importance

Chemically, imidazo[1,2-*a*]pyridines are highly valued in synthetic organic chemistry and catalysis. Their two nitrogen atoms provide strong coordination ability toward transition metals, enabling the design of metal–ligand complexes for various catalytic applications.^120^ They can participate in C–H activation, N-alkylation, cyclization, and multicomponent reactions, making them crucial intermediates for constructing diverse heterocyclic architectures.^121^ The Groebke–Blackburn–Bienaymé Reaction (GBBR), for instance, is a common synthetic route to access numerous imidazo[1,2-*a*]pyridine derivatives.^122^

##### 1.3.6.1. Chemical examples

Imidazo[1,2-*a*]pyridine–Pd(ii) complexes – catalyze suzuki, heck, and sonogashira coupling reactions with high efficiency.^123^

Imidazo[1,2-*a*]pyridine–Schiff base ligands – used in oxidation and hydrogenation catalysis.^123^

Fluorescent imidazo[1,2-*a*]pyridine probes – detect heavy metal ions such as Cu^2+^, Hg^2+^, and Fe^3+^.^124^

GBBR-derived analogs – applied in combinatorial chemistry to synthesize drug-like libraries.^125^

These chemical attributes highlight the synthetic versatility and coordination potential of the imidazo[1,2-*a*a]pyridine nucleus.

As a result, imidazo[1,2-*a*]pyridine stands out as a multifunctional heterocyclic framework bridging industrial innovation, biological activity, and medicinal utility. Its robust chemical stability, structural flexibility, and wide pharmacological range have made it a cornerstone in contemporary research.^126^ From industrial catalysis and luminescent materials to life-saving pharmaceuticals and eco-friendly agrochemicals, this scaffold continues to evolve as a universal molecular platform with extraordinary interdisciplinary relevance.

### 1.4. Background on synthesis of imidazo[1,2-*a*]pyridines

The synthesis of imidazo[1,2-*a*]pyridines has been an active area of research in heterocyclic chemistry for several decades due to the structural and pharmacological importance of this bicyclic scaffold. Historically, the first imidazo[1,2-*a*]pyridines were synthesized in the early 20th century through condensation reactions between 2-aminopyridine and α-haloketones, a method that remains one of the most widely used and reliable routes.^117,127^ This classical approach, often referred to as the Chichibabin or Debus-type synthesis, involves cyclization *via* nucleophilic substitution, where the amino group of 2-aminopyridine attacks the carbonyl carbon of the haloketone to form the fused imidazo ring system.^118^ The reaction typically proceeds under mild heating or reflux conditions and provides moderate to high yields of the desired heterocycle.

Over the years, numerous modifications and alternative synthetic routes have been developed to improve efficiency, substrate diversity, and environmental sustainability. Among these, the multicomponent reaction (MCR) approach has gained significant prominence. A notable example is the Groebke–Blackburn–Bienaymé reaction (GBBR), which combines 2-aminopyridine, an aldehyde, and an isonitrile in a single-step, one-pot process.^128^ This reaction offers a rapid and atom-economical route to highly substituted imidazo[1,2-*a*]pyridines under mild conditions, making it particularly valuable for combinatorial and medicinal chemistry applications. The MCR strategy allows for easy structural variation at multiple ring positions, facilitating the synthesis of diverse libraries of derivatives for biological screening.

Recent research on the synthesis of imidazo[1,2-*a*]pyridines has expanded beyond classical and multicomponent approaches to emphasize greener, catalyst-driven methodologies. Efforts now commonly feature metal-assisted cyclizations, energy-efficient techniques such as microwave or ultrasound activation, and environmentally conscious conditions using solvent-free systems or ionic liquids. Transition metals, including copper, palladium, iron, and zinc, have been explored for their ability to facilitate key bond-forming steps with notable selectivity, while microwave and ultrasonic technologies help reduce reaction times and improve overall efficiency. In parallel, the emergence of nanocatalysts and other heterogeneous systems offers recyclable, high-performance options that further support the sustainable synthesis of imidazo[1,2-*a*]pyridine derivatives.

In summary, the synthesis of imidazo[1,2-*a*]pyridines has evolved from traditional condensation reactions to advanced catalytic and multicomponent strategies, reflecting the continuous pursuit of more sustainable, efficient, and versatile methods. The availability of diverse synthetic pathways enables chemists to design tailored derivatives with specific physical, electronic, or biological properties. Consequently, the imidazo[1,2-*a*]pyridine framework remains a cornerstone in heterocyclic synthesis, bridging classical organic chemistry with modern green and medicinal chemistry approaches.

## 2. Magnetic nanocatalysts in synthesis of imidazo[1,2-*a*]pyridines

Magnetic nanocatalysts (MNCs) have emerged as remarkably efficient, sustainable, and reusable catalytic systems, revolutionizing the synthesis of imidazo[1,2-*a*]pyridine derivatives.^129^ These derivatives represent essential heterocyclic frameworks that play a pivotal role in both medicinal and industrial chemistry, offering innovative pathways for drug development and chemical manufacturing. The unique properties of MNCs not only enhance reaction rates but also contribute to a greener approach by minimizing waste and maximizing efficiency in synthetic processes. These catalysts typically consist of magnetic nanoparticles coated or functionalized with suitable catalytic species, including transition metals, organic ligands, or acidic/basic groups.^130^ Their nanoscale dimensions provide a large specific surface area, high active site density, and excellent electron transfer ability, leading to enhanced catalytic performance. In reactions such as the cyclocondensation of 2-aminopyridines with aldehydes and isonitriles (Groebke–Blackburn–Bienaymé reaction) or with α-haloketones, magnetic nanocatalysts act as Lewis acid sites that activate the carbonyl or imine groups, accelerating the formation of the imidazo ring system under mild and green conditions. Magnetic nanocatalysts offer a key advantage in that they can be rapidly separated and recovered with an external magnet, avoiding labor-intensive processes such as filtration or centrifugation. Their robust stability and ability to be reused across many catalytic runs with minimal activity loss further support their value in sustainable chemistry. By merging the strengths of both heterogeneous and homogeneous catalysts—high efficiency, strong selectivity, and straightforward recyclability—these systems provide an attractive platform for green synthesis, particularly for nitrogen-rich heterocycles like imidazo[1,2-*a*]pyridines.

### 2.1. Scope of the review

This review aims to provide a comprehensive overview of the chemical, biological, and industrial significance of imidazo[1,2-*a*]pyridine derivatives, with a particular emphasis on the role of magnetic nanocatalysts in their synthesis. It highlights the structural versatility, pharmacological importance, and multifunctional applications of this heterocyclic scaffold across pharmaceutical, agrochemical, and material science fields. The review systematically discusses the traditional and modern synthetic routes used for constructing imidazo[1,2-*a*]pyridines, including classical condensation reactions, multicomponent approaches, and green catalytic methods. Particular emphasis is placed on the rise of magnetic nanocatalysts (MNCs) as sustainable, efficient, and reusable systems that facilitate eco-friendly, high-yield synthetic transformations. Furthermore, the article explores recent advancements in the design, characterization, and mechanistic role of Fe_3_O_4_-based and composite nanocatalysts in promoting imidazo[1,2-*a*a]pyridine formation through one-pot and multicomponent reactions. Comparative discussions are presented between conventional catalysts and nanostructured magnetic systems in terms of efficiency, selectivity, stability, and reusability. By consolidating these developments, this review seeks to underline the growing importance of nanotechnology in heterocyclic synthesis and to provide a scientific foundation for future research directed toward the eco-friendly and scalable production of imidazo[1,2-*a*]pyridine frameworks with enhanced catalytic and functional performance.

### 2.2. Catalysis by magnetic nanoparticles

Magnetic nanoparticles have emerged as highly effective heterogeneous catalysts for heterocycle construction, owing to their large surface area and tunable surfaces, which enhance the activation of reactants.^131,132^ They also offer notable practical benefits, as their magnetic responsiveness enables swift post-reaction recovery and repeated reuse with minimal loss of performance.^133–135^ Catalysis by magnetic nanoparticles enables reactions to run under milder conditions by providing highly active, nanoscale surfaces that accelerate bond-breaking and bond-forming steps, reducing the need for strong acids, bases, or high pressures. Their efficient heat and mass transfer, combined with tunable surface functionalization, often lowers the reaction temperature required for high conversion because reactants interact more readily with catalytic sites and activate at lower thermal energy. Recent investigations have demonstrated their successful application in the synthesis of imidazo[1,2-*a*]pyridines, highlighting both their catalytic versatility and their potential to advance the development of this important class of compounds.

**Scheme 1:**
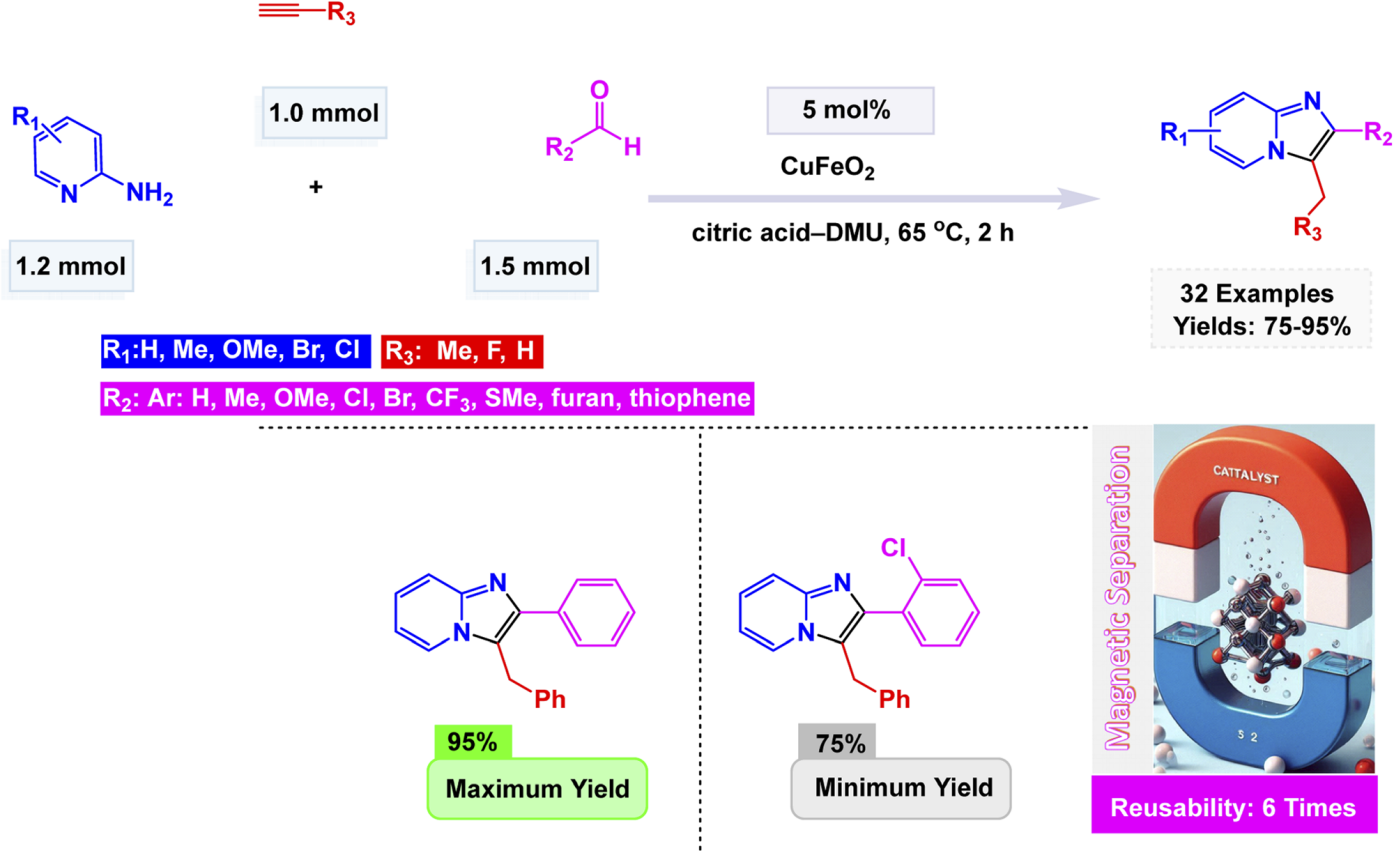
CuFeO_2_ nanoparticles catalyzed the synthesis of imidazo[1,2-*a*]pyridines.

Zhang *et al.* unveiled a remarkable advancement in nanotechnology with the synthesis of CuFeO_2_ nanoparticles through a hydrothermal method.^136^ These groundbreaking nanoparticles emerge as a remarkably efficient and recyclable nanocatalyst, streamlining the eco-friendly synthesis of imidazo[1,2-*a*]pyridines. This remarkable transformation unfolds through a seamless one-pot, three-component reaction that artfully combines 2-aminopyridines, aldehydes, and alkynes, all while harnessing a citric acid–dimethylurea (DMU) melt as a sustainable green solvent at 65 °C for 2 h. The morphology of these nanoparticles was meticulously analyzed, with Scanning Electron Microscopy (SEM) and Transmission Electron Microscopy (TEM) offering revealing insights. The CuFeO_2_ nanoparticles exhibit a beautifully uniform, spherical shape, with an average diameter ranging from 30 to 35 nanometers (nm). This precise measurement aligns flawlessly with calculations derived from Scherrer's equation, reinforcing the accuracy and reliability of the findings. As depicted in Scheme 1, a wide variety of aldehydes, whether adorned with electron-donating groups or subtly electron-withdrawing ones, engaged effortlessly with pyridin-2-amine and ethynylbenzene. This synergy resulted in the successful formation of the desired products, showcasing impressively high to excellent yields. Remarkably, the CuFeO_2_ catalyst exhibited exceptional stability, with its catalytic performance remaining steadfast even after reuse up to six times, emphasizing its tremendous potential for sustainable applications in organic synthesis.

**Scheme 2:**
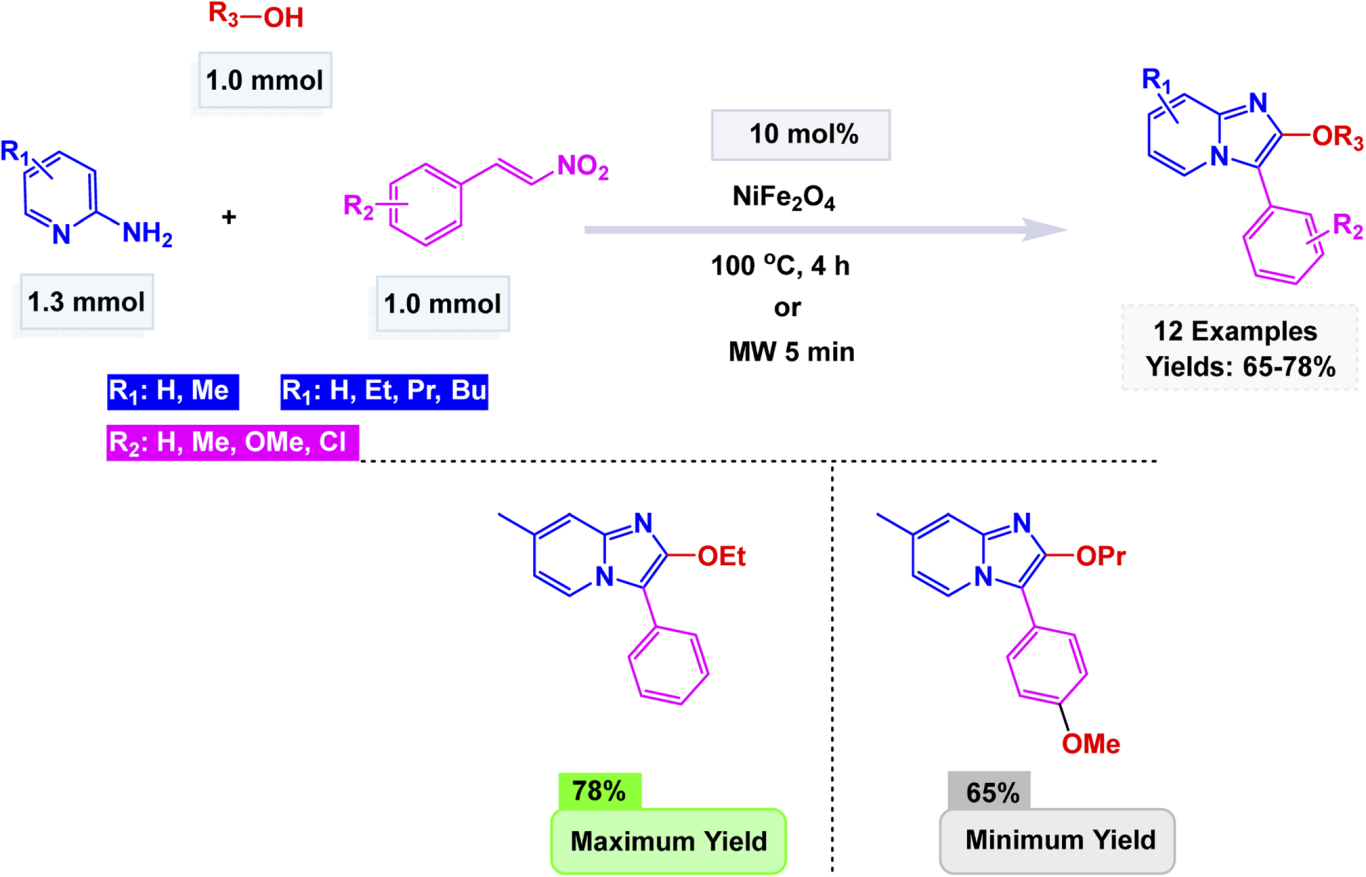
CuFeO_2_ nanoparticles catalyzed the synthesis of imidazo[1,2-*a*]pyridines.

**Scheme 3:**
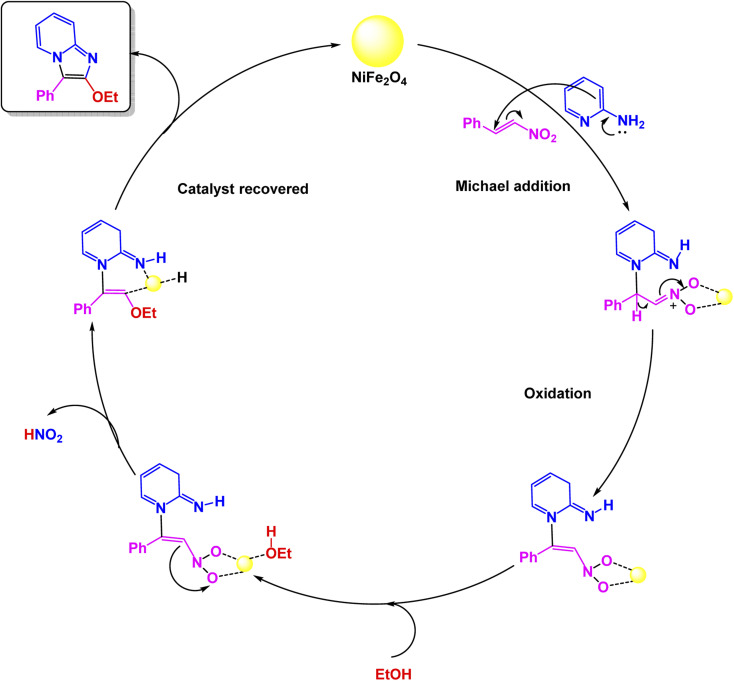
Plausible mechanism for CuFeO_2_ nanoparticles catalyzed synthesis of imidazo[1,2-*a*]pyridines.

In the same context, Banerjee *et al.* developed a magnetically recoverable NiFe_2_O_4_ spinel nanocatalyst prepared *via* a sol–gel process, whose cubic spinel structure was confirmed by X-ray Diffraction (XRD), High-Resolution Transmission Electron Microscopy (HRTEM), and Field-Emission Scanning Electron Microscopy (FESEM), showing uniform spherical particles of approximately 15 nm.^137^ The catalyst was evaluated in the three-component synthesis of 2-alkoxy-3-arylimidazo[1,2-*a*]pyridines under two distinct conditions: conventional heating (100 °C for 4 h) and microwave irradiation Scheme 2. The proposed pathway Scheme 3 involves an aza-Michael addition followed by oxidative imination and nitro group elimination, furnishing C-2 alkoxylated imidazo[1,2-*a*]pyridines. Although both methods afforded the target products efficiently, the microwave-assisted protocol proved superior, offering significantly reduced reaction time 5 minutes (min) and cleaner conversion compared to the thermally heated system. The NiFe_2_O_4_ catalyst exhibited excellent magnetic separability and was reused for eight consecutive cycles with negligible loss of activity or change in structure, as confirmed by Fourier Transform Infrared Spectroscopy (FT-IR), XRD, and TEM. Overall, this study demonstrated that NiFe_2_O_4_ acts as an effective and recyclable spinel ferrite nanocatalyst, providing a fast, green, and solvent-free route for the synthesis of 2-alkoxy-3-arylimidazo[1,2-*a*]pyridines.

**Scheme 4:**
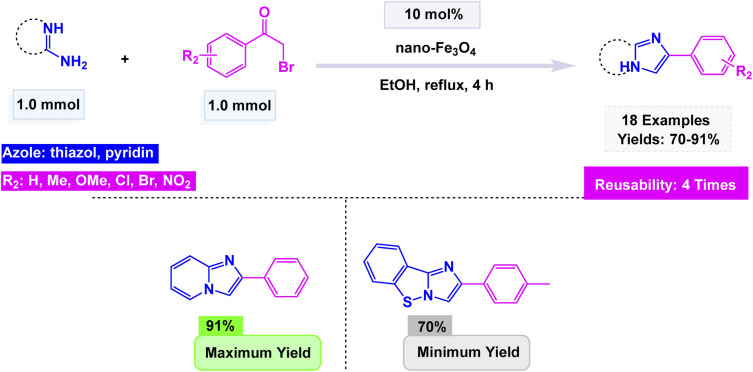
Fe_3_O_4_ nanoparticles catalyzed the synthesis of imidazo[1,2-*a*]pyridines.

Chaudhari *et al.* synthesized nano-Fe_3_O_4_ by a co-precipitation method using Fe^2+^ and Fe^3+^ salts in an alkaline medium and characterized it by FT-IR, XRD, and SEM, confirming the crystalline magnetite structure with nanoscale morphology.^138^ The catalyst displayed astonishing efficiency in facilitating the condensation reaction between 2-aminopyridine, 2-aminothiazole, and 2-aminobenzothiazole at reflux in ethanol for 4 h, interacting seamlessly with a diverse range of substituted phenacyl bromides. Conducted under the gentle embrace of refluxing ethanol's warmth, this remarkable process gave rise to a stunning variety of imidazo[1,2-*a*]pyridines, achieving yields that soared to an impressive 91%. The vibrant interplay of these components painted a vivid picture of chemical transformation at its finest illustrated in Scheme 4. Notably, the catalyst demonstrated the unique ability to be magnetically recovered, allowing for its reuse over four consecutive runs with only a minimal decline in activity. In comparative studies, its performance overshadowed that of other ferrite catalysts, including NiFe_2_O_4_, CuFe_2_O_4_, MnFe_2_O_4_, and ZnFe_2_O_4_, under identical experimental conditions, establishing itself as a superior choice for these transformations.

**Scheme 5:**
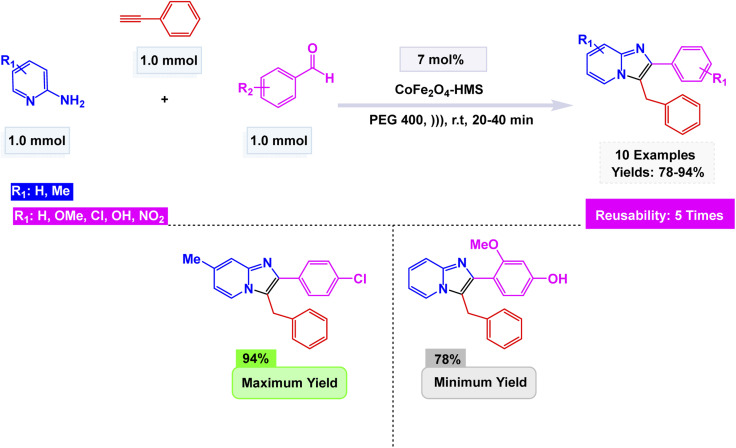
CoFe_2_O_4_–HMS-catalyzed ultrasonic-assisted A^3^-coupling synthesis of imidazo[1,2-*a*]pyridine derivatives.

**Scheme 6:**
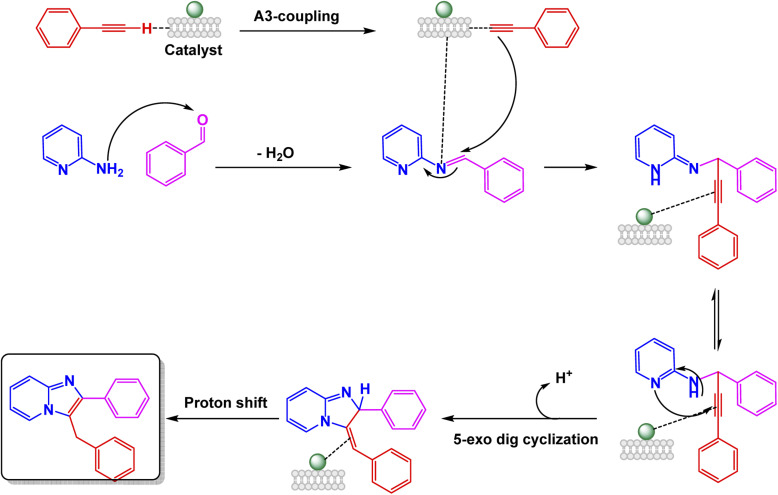
Plausible mechanistic pathway for CoFe_2_O_4_–HMS-catalyzed ultrasonic-assisted A^3^-coupling synthesis of imidazo[1,2-*a*]pyridine derivatives.

Geedkar and colleagues crafted an innovative and environmentally friendly catalyst that showcases the remarkable properties of cobalt ferrite nanoparticles elegantly supported on hydromagnesite sheets (CoFe_2_O_4_–HMS). This cutting-edge catalyst is not only magnetically recoverable but also plays a pivotal role in the ultrasonic-assisted synthesis of imidazo[1,2-*a*]pyridine derivatives, making it a standout in the realm of sustainable chemistry.^101^ The nanocomposite, prepared by co-precipitation and characterized by XRD, FE-SEM, HR-TEM, FT-IR, Raman, Brunauer–Emmett–Teller (BET), Carbon Dioxide Temperature-Programmed Desorption (CO_2_-TPD), and Vibrating Sample Magnetometry (VSM), exhibited a crystalline spinel structure, a mesoporous surface area of 75 m^2^ g^−1^, and a saturation magnetization of 20.16 emu g^−1^, ensuring easy magnetic separation. The A^3^-coupling of 2-aminopyridine, aryl aldehydes, and phenylacetylene unfolded seamlessly in the vibrant medium of Polyethylene Glycol 400 (PEG-400) at room temperature, energized by the rhythmic pulse of sonication. This dynamic process led to remarkable yields, soaring up to an impressive 94%, all achieved within remarkably brief reaction times. The efficiency and speed of this reaction highlight the potential for innovation in synthetic chemistry Scheme 5. The mechanistic pathway Scheme 6 involves iminium formation, cobalt–acetylide generation, and subsequent 5-*exo*-dig cyclization to form the fused heterocycle. The catalyst maintained excellent activity for five consecutive cycles with negligible loss of efficiency or structural change, demonstrating its potential as a green, durable, and high-performance spinel ferrite system for sustainable heterocyclic synthesis.

**Scheme 7:**
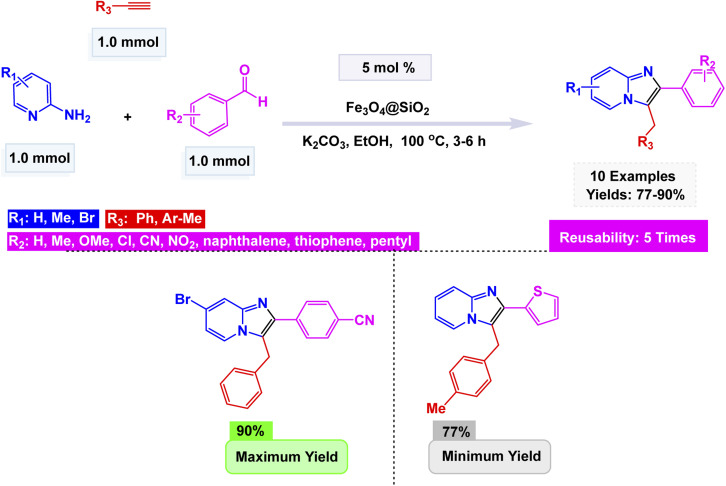
Fe_3_O_4_@SiO_2_ NPs catalyzed the synthesis of imidazo[1,2-*a*]pyridines.

In line with these findings, Maleki introduced an efficient Fe_3_O_4_@SiO_2_ nanocatalyst for the one-pot multicomponent synthesis of imidazo[1,2-*a*]pyridines.^102^ The catalyst was prepared by coating Fe_3_O_4_ nanoparticles with silica (SiO_2_) *via* a sol–gel process, affording uniform spherical nanostructures with excellent thermal and magnetic stability. When subjected to carefully optimized conditions, the reaction between 2-aminopyridine, an aldehyde, and a terminal alkyne unfolded seamlessly in ethanol at 100 °C for 3–6 h. The dynamic blend was enhanced by a subtle catalyst: a mere 5 mol% of Fe_3_O_4_@SiO_2_ combined with K_2_CO_3_. This harmonious combination led to the successful formation of a rich array of imidazo[1,2-*a*]pyridine derivatives, showcasing the elegant interplay of these reagents as they transformed under the perfect conditions Scheme 7. The reaction mechanism involves initial imine formation, followed by nucleophilic attack of the alkyne carbanion, intramolecular cyclization, and isomerization to yield the fused heterocycle. The catalyst exhibited outstanding efficiency, surpassing the performance of Fe_3_O_4_ and SiO_2_ when used individually. Remarkably, it showcased the ability to be magnetically recovered and reused, maintaining its impressive activity over five cycles with minimal loss.

The catalytic systems reported display clear structure–property–activity relationships driven by nanoparticle size, surface area, and reaction conditions Table 2. CuFeO_2_ nanoparticles, with a relatively small size of 30–35 nm and a high BET surface area of 118.6 m^2^ g^−1^, performed efficiently at mild conditions (citric acid–DMU, 65 °C, 2 h), enabling 32 examples with high yields (75–95%) and good reusability over six cycles (Entry 1). The combination of moderate nanoparticle size and high surface area appears to support strong adsorption and activation of substrates under low-temperature conditions. In contrast, NiFe_2_O_4_ system (15 nm) required harsher thermal conditions (100 °C, 4 h) to achieve modest yields (65–78%) despite the smaller particle size; however, microwave irradiation (5 min) significantly reduced time but maintained similar yields, indicating that NiFe_2_O_4_ benefits more from rapid dielectric heating than from conventional reflux (Entry 2). Nano-Fe_3_O_4_ (60 ± 15 nm), used under ethanolic reflux for 4 h, produced higher yields (70–91%) across 18 examples but showed moderate reusability (four cycles), suggesting that the larger particle size and lower surface exposure reduce catalytic efficiency, requiring longer reaction times and solvent heating to achieve competitive performance (Entry 3). Finally, CoFe_2_O_4_–HMS composite, characterized by crystallite sizes of 12.30–16.97 nm and a BET surface area of 75 m^2^ g^−1^, performed exceptionally well under green conditions (PEG-400, 20–40 min), providing high yields (78–94%) with five reuse cycles (Entry 4). The synergistic effect of encapsulating CoFe_2_O_4_ in a high-area mesoporous HMS scaffold likely enhances dispersion and stabilizes active sites, enabling reactions to proceed rapidly at low viscosity and without classical organic solvents. Overall, the comparison indicates that surface area and nano-dispersion strongly influence catalytic efficiency, while reaction medium and thermal input determine energy efficiency and operational practicality.

**Table 2:** Quantitative comparison of magnetic nanocatalysts used for imidazo[1,2-*a*]pyridine synthesis

NO.	Cat. a		Size (nm)	SBET^b^	Rxn. Cond.^c^	*T* d	Yields (%)	Expt.^e^	Cycles
1		CuFeO_2_	30–35 nm (TEM)	118.6 m^2^ g^−1^	Citric acid–DMU, 65 °C	2 h	75–95	32	6
2		NiFe_2_O_4_	15 nm (TEM)	—	100 °C, 4 h or MW 5 min	4 h	65–78	12	8
3		Fe_3_O_4_	60 ± 15 nm (SEM)	—	EtOH, reflux	4 h	70–91	18	4
4		CoFe_2_O_4_–HMS	12.30–16.97 nm (XRD)	75 m^2^ g^−1^	PEG-400, sonication	20–40 min	78–94	10	5

a Catalyst.

b BET surface area (m^2^ g^−1^).

c Reaction conditions.

d Temperature (°C).

e Number of examples tested.

### 2.3. Catalysis by magnetic nanoparticles supported copper catalysts

Copper catalysts play a crucial role in many chemical reactions due to copper's versatile oxidation states, ability to coordinate with a wide range of ligands, and strong catalytic activity in both organic and inorganic transformations.^139,140^ They facilitate processes such as oxidation, reduction, coupling reactions, and click chemistry, often providing high selectivity and efficiency under relatively mild conditions.^141^ Copper catalysts can be used in homogeneous forms, like copper salts and complexes, or heterogeneous forms, such as supported copper nanoparticles or oxides, which offer advantages in stability and recyclability.^142^ Because they are generally inexpensive, abundant, and environmentally friendlier than many noble-metal catalysts, copper-based systems are widely applied in industrial synthesis, pharmaceuticals, materials science, and green chemistry methodologies.

A key benefit of magnetic nanoparticle–supported copper catalysts is their easy and rapid recovery from the reaction medium using an external magnet, eliminating traditional, more labor-intensive purification steps.^143^ This magnetic separability, coupled with the typically low cost and abundance of copper, makes these catalysts particularly appealing for sustainable and economically practical synthesis. Additionally, their reusability reduces waste and contributes to greener chemical processes.^144,145^ Overall, they offer a powerful approach for developing recyclable, high-performance catalytic systems suitable for both academic research and industrial applications.^30,146^ In recent studies, multiple research teams have harnessed the power of magnetic nanoparticles supported copper catalysts to synthesize imidazo successfully [1,2-*a*]pyridines.

**Scheme 8:**
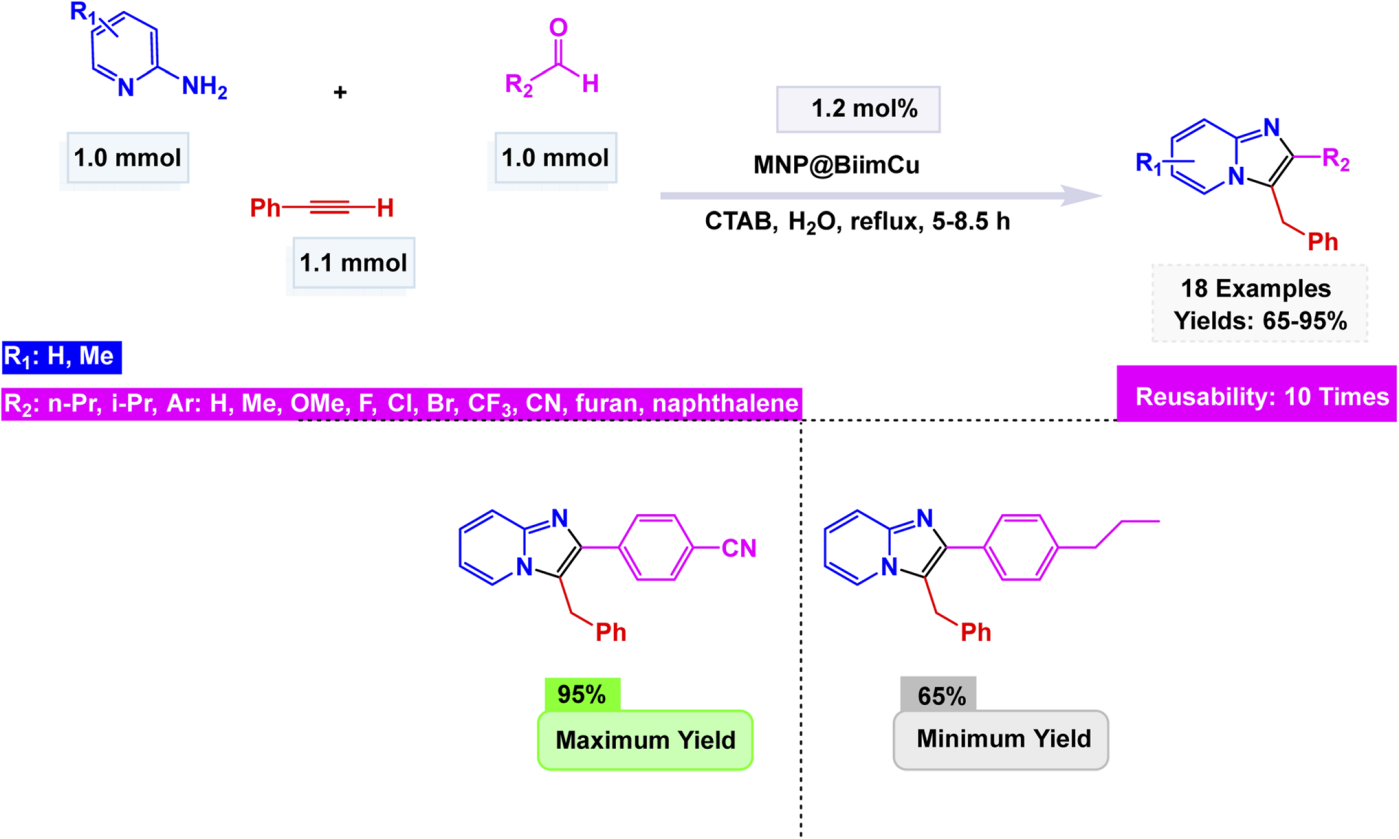
MNP@BiimCu nanocomposite catalyzed synthesis of imidazo[1,2-*a*]pyridines.

Tajbakhsh and colleagues meticulously crafted a magnetically recoverable nano-catalyst by covalently attaching biimidazole (Biim) to chloride-functionalized silica@magnetite nanoparticles (MNP). This innovative process was artfully followed by metalation using CuI, resulting in the formation of a striking biimidazole Cu(i) complex.^147^ Electron microscopy investigations, including SEM and TEM, revealed that these nanoparticles exhibit a predominantly spherical morphology, measuring an average of just 20 nm in diameter. The MNP@BiimCu nanocomposite was brilliantly utilized in the synthesis of imidazo[1,2-*a*]pyridines, adeptly facilitating the transformation of a wide variety of both aliphatic and aromatic aldehydes through dynamic interactions with 2-aminopyridine and phenylacetylene. This cutting-edge method yielded striking results, as vividly illustrated in the accompanying Scheme 8. Among the most remarkable outcomes were the reactions involving 5-methyl-2-aminopyridine and 3-methyl-2-aminopyrimidine with benzaldehyde, which produced the sought-after imidazo[1,2-*a*]pyridines in impressive yields of 82% and 78%, respectively. However, attempts to incorporate dimethylamino or nitro substituents on the aromatic rings of the aldehydes presented significant challenges, completely halting the reactions and resulting in no products whatsoever. The MNP@BiimCu catalyst, easily recoverable through the simple application of a magnet, has demonstrated exceptional efficiency and durability, having been successfully reused for at least 10 consecutive runs without any noticeable decline in catalytic activity.

**Scheme 9:**
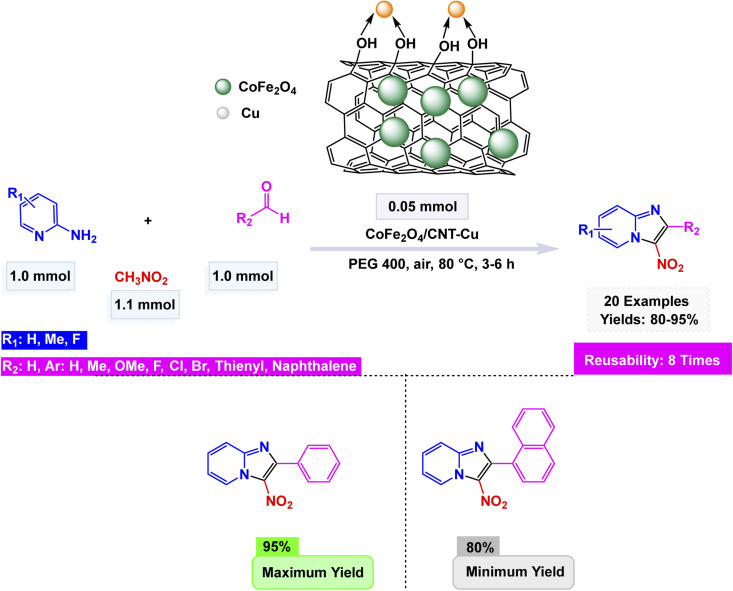
CoFe_2_O_4_/CNT–Cu catalyzed synthesis of 3-nitro-2-arylimidazo[1,2-*a*]pyridines.

**Scheme 10:**
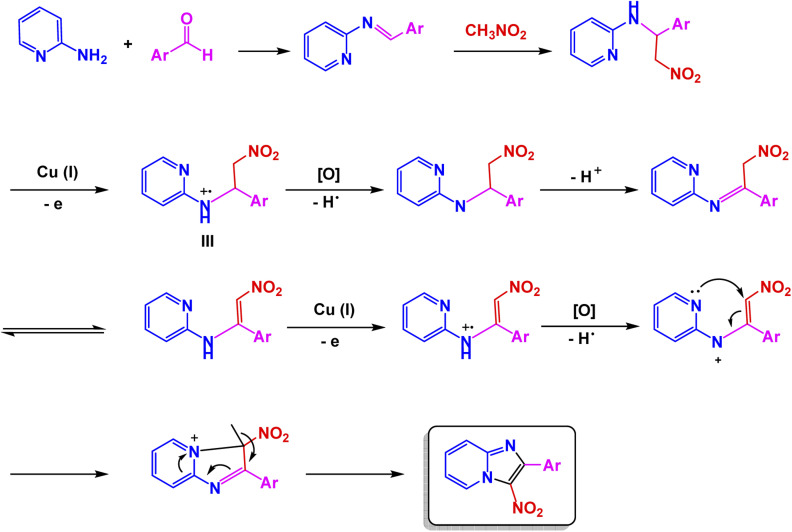
Proposed mechanism for CoFe_2_O_4_/CNT–Cu catalyzed formation of 3-nitro-2-arylimidazo[1,2-*a*]pyridines.

Zhan-Hui Zhang and co-workers developed a magnetically recoverable nanocatalyst, CoFe_2_O_4_/CNT–Cu, composed of copper species supported on cobalt ferrite-decorated carbon nanotubes (CNT).^148^ Characterization by XRD, FT-IR, SEM, TEM, BET, VSM, and Energy-Dispersive X-ray Spectroscopy (EDS) confirmed a crystalline spinel structure with uniformly dispersed spherical particles (50 nm), a surface area of 26.7 m^2^ g^−1^, and superparamagnetic behavior (21.8 emu g^−1^). The catalyst demonstrated remarkable reactivity in the one-pot three-component synthesis of 3-nitro-2-arylimidazo[1,2-*a*]pyridines, skillfully facilitating the coupling of 2-aminopyridine, aromatic aldehydes, and nitromethane in the versatile solvent PEG-400 at a controlled temperature of 80 °C. This efficient process yielded impressive results, with product formation reaching between 80% and 95% using only 5 mol% of the catalyst see Scheme 9 for details. The reaction exhibited a broad tolerance for various substituents, accommodating both electron-donating and electron-withdrawing groups with ease. However, it was noted that sterically hindered aldehydes tended to yield slightly lower results, showcasing the sensitivity of the reaction to steric effects. The proposed mechanism Scheme 10 begins with condensation of 2-aminopyridine and the aldehyde to form an imine intermediate (I), followed by the Michael-type addition of nitromethane to yield the β-nitroamine adduct (II). Intramolecular cyclization and oxidative aromatization, facilitated by surface Cu(ii) species, lead to the formation of the 3-nitro-2-arylimidazo[1,2-*a*]pyridine. The CoFe_2_O_4_/CNT–Cu nanocatalyst showcased extraordinary magnetic separability and remarkable reusability across eight consecutive cycles, maintaining its catalytic performance and demonstrating exceptional stability, all while contributing to environmentally friendly practices in sustainable heterocyclic synthesis.

**Scheme 11:**
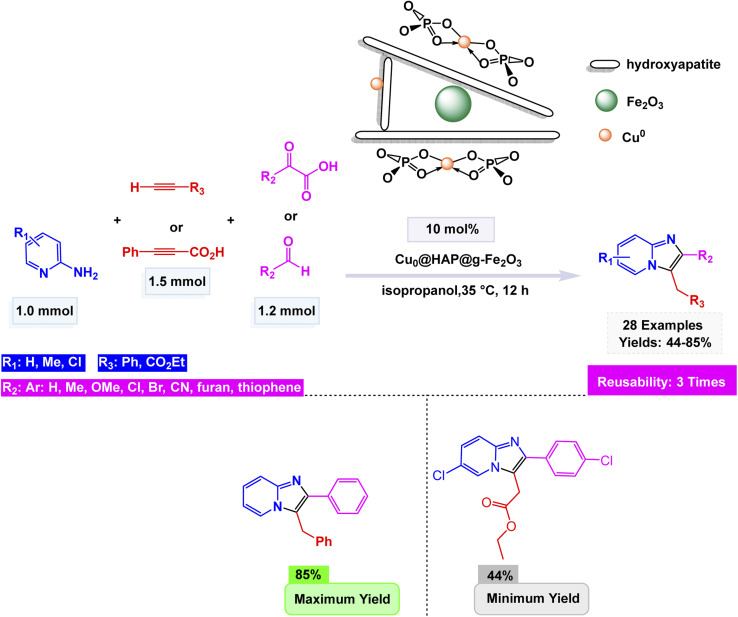
Cu^0^@HAP@γ-Fe_2_O_3_-catalyzed synthesis of imidazo[1,2-*a*]pyridines.

Likewise, Yiqun Li and co-workers synthesized a magnetic hybrid nanocatalyst, Cu^0^@HAP@γ-Fe_2_O_3_, by sequential ion-exchange, oxidation, and reduction steps to immobilize metallic copper nanoparticles onto hydroxyapatite-encapsulated maghemite (HAP).^149^ The composite was thoroughly characterized by FT-IR, XRD, SEM, TEM, EDS, X-ray Photoelectron Spectroscopy (XPS), Inductively Coupled Plasma – Atomic Emission Spectroscopy (ICP-AES), and Thermogravimetric Analysis (TGA), revealing a crystalline hybrid structure with uniformly distributed Cu^0^ species (40 nm), good thermal stability, and efficient metal dispersion. The nanocatalyst (10 mol%) demonstrated excellent activity in a one-pot three-component coupling of 2-aminopyridine, aromatic aldehydes (or phenylglyoxylic acids), and terminal alkynes (or alkynyl carboxylic acids) to afford imidazo[1,2-*a*]pyridine derivatives Scheme 11. The plausible mechanism involves the initial formation of an imine between 2-aminopyridine and the aldehyde (or glyoxylic acid), followed by a copper-assisted alkyne activation, nucleophilic addition, and oxidative cyclization through a decarboxylative or oxidative C–N coupling pathway to yield the fused heterocycle. The catalytic system displayed remarkable versatility, effortlessly accommodating a diverse range of substituents, both electron-donating and electron-withdrawing. It even embraced the complexity of heteroaromatic aldehydes, showcasing its adaptive prowess in the face of varied chemical landscapes. Furthermore, the use of alkynyl or glyoxylic acids enabled a dual-decarboxylative variant, expanding substrate scope. The Cu^0^@HAP@γ-Fe_2_O_3_ catalyst showcased remarkable magnetic properties, allowing it to be effortlessly separated and reclaimed. It was put to the test across three successive cycles, demonstrating only a minimal decline in activity, highlighting its impressive durability and resilience.

**Scheme 12:**
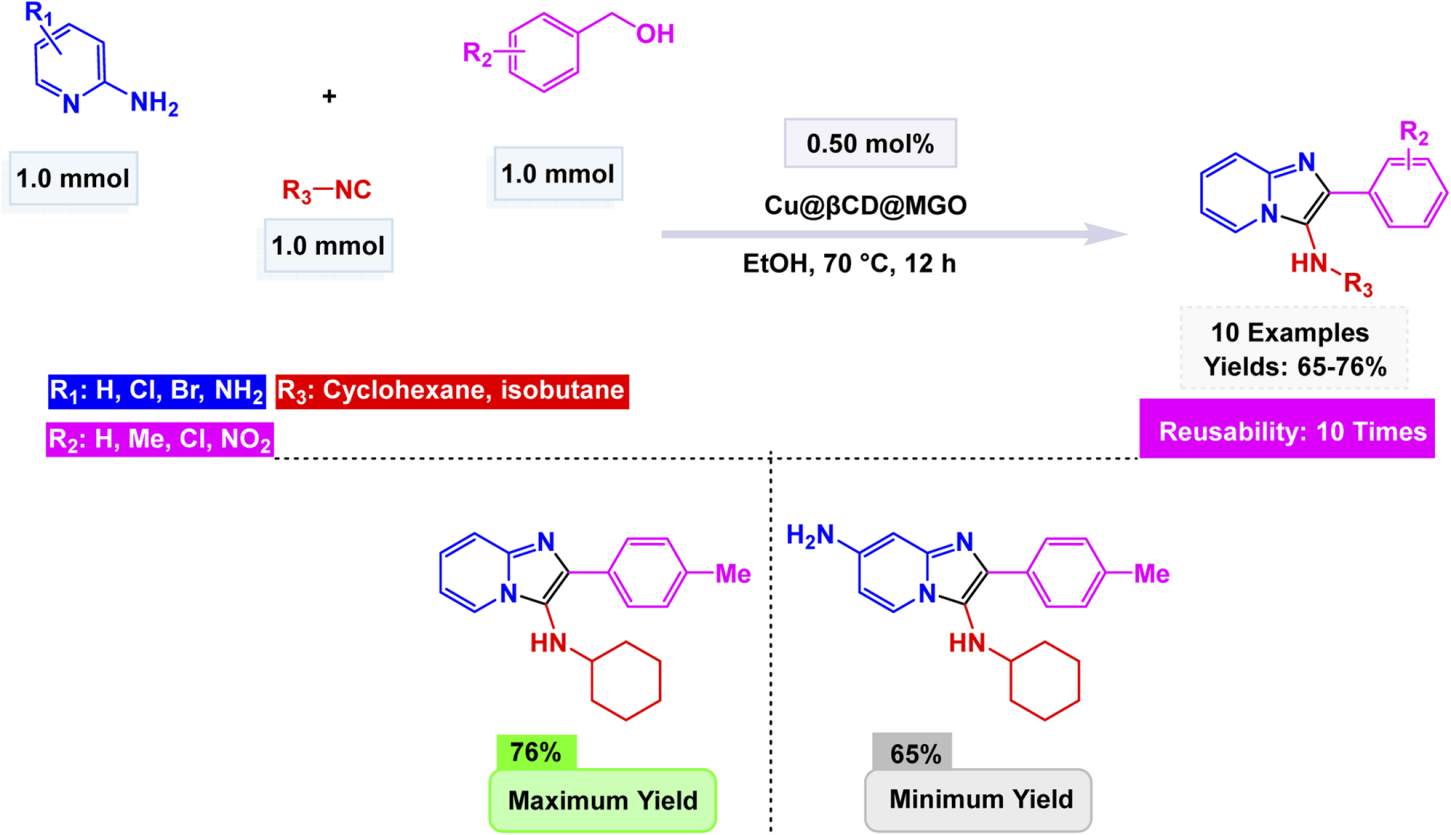
Cu@β-CD@MGO synthesis of N-alkyl-2-phenylimidazo[1,2-*a*]pyridin-3-amines.

In continuation, Bahadorikhalili *et al.* designed a multifunctional copper-based nanocatalyst, Cu@β-CD@MGO, prepared by immobilizing Cu species onto β-cyclodextrin (β-CD)–functionalized magnetic graphene oxide nanosheets (MGO).^150^ The Cu@β-CD@MGO catalyst underwent an extensive characterization through various spectroscopic analyses, which revealed a uniform dispersion of copper nanoparticles throughout the material. These nanoparticles exhibited a mean size ranging between 20 and 25 nanometers, showcasing their finely tuned dimensions. Additionally, the catalyst demonstrated superparamagnetic properties, allowing for effortless recovery and handling, making it both efficient and practical for various applications. This innovative system adeptly facilitated a remarkable one-pot oxidative three-component reaction, seamlessly integrating benzyl alcohols, 2-aminopyridines, and isocyanides under aerobic conditions to yield phenylimidazo[1,2-*a*]pyridine in impressive amounts see Scheme 12. The proposed mechanism unfolds with the initial aerobic oxidation of benzyl alcohol, transforming it into the corresponding aldehyde through the proficient catalysis of Cu@β-CD@MGO. This step is swiftly followed by the condensation with 2-aminopyridine, leading to the formation of a tantalizing imine intermediate, setting the stage for further transformations in this intricate synthetic journey. Subsequent attack of the isocyanide, intramolecular cyclization, and 1,3-hydride shift afford the desired imidazopyridine derivative. The methodology tolerates both electron-rich and electron-deficient substrates, demonstrating broad applicability. Notably, the catalyst was magnetically separable and reused for ten successive runs without appreciable copper leaching or reduction in activity, as confirmed by ICP-AES and VSM analyses, emphasizing its robustness, high turnover number (TON ≈ 1500), and green efficiency in multicomponent heterocyclic synthesis.

**Scheme 13:**
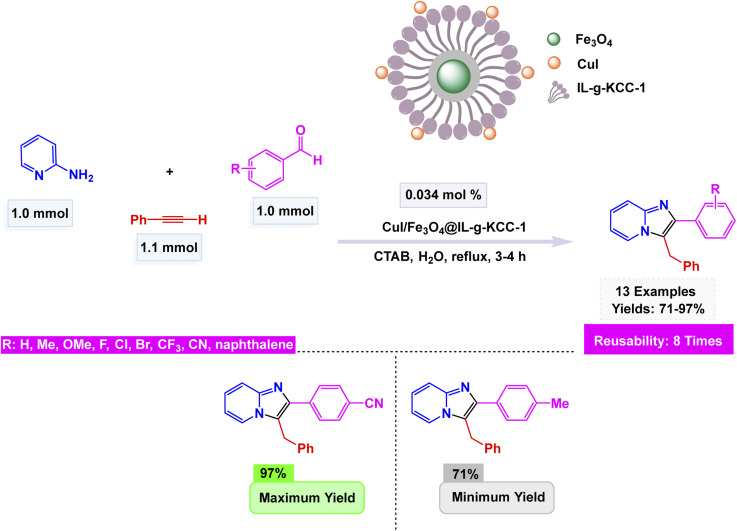
CuI/Fe_3_O_4_ NPs@Biimidazole IL–KCC-1-catalyzed synthesis of imidazo[1,2-*a*]pyridines.

Shadjou and co-workers synthesized a novel leach-proof magnetic nanocatalyst, CuI/Fe_3_O_4_ NPs@Biimidazole IL–KCC-1, by anchoring Fe_3_O_4_ nanoparticles and CuI salts onto dendritic fibrous nanosilica (KCC-1) functionalized with a biimidazole ionic liquid.^151^ The SEM and TEM images showcase a stunning dendritic morphology, where Fe_3_O_4_ and CuI nanoparticles are elegantly distributed throughout the structure. These nanoparticles boast an average size of 30 nm, exhibiting a captivating superparamagnetic behavior. Remarkably, they demonstrate a magnetization value of 22.47 emu g^−1^, a finding that has been substantiated through VSM analysis. The catalyst demonstrated exceptional efficacy in the elegant one-pot three-component synthesis of imidazo[1,2-*a*]pyridines, achieved by condensing 2-aminopyridine, aromatic aldehydes, and phenylacetylene in water under diligent reflux conditions. This meticulous process yielded remarkable products with impressive efficiencies ranging from 71 to 97% see Scheme 13. The proposed mechanism unfolds like a well-orchestrated symphony, beginning with the formation of an imine between 2-aminopyridine and the aldehyde. This is swiftly followed by the creation of a copper-acetylide complex, which then embarks on a nucleophilic adventure, attacking the carbon of the imine. This critical step catalyzes a cyclization reaction, ultimately crafting the intricate imidazo[1,2-*a*]pyridine framework. The system displayed a remarkable tolerance for a diverse array of substituents, revealing that electron-withdrawing groups such as Cl, Br, CN, F, and CF_3_ significantly enhanced the yields compared to their electron-donating counterparts. Notably, the catalyst is not just a one-time wonder; it demonstrated its prowess as a magnetically recoverable entity, successfully being reused across eight consecutive cycles with negligible loss of its catalytic activity. This resilience underscores the robust metal–support interaction and resistance to leaching inherent in the system.

**Scheme 14:**
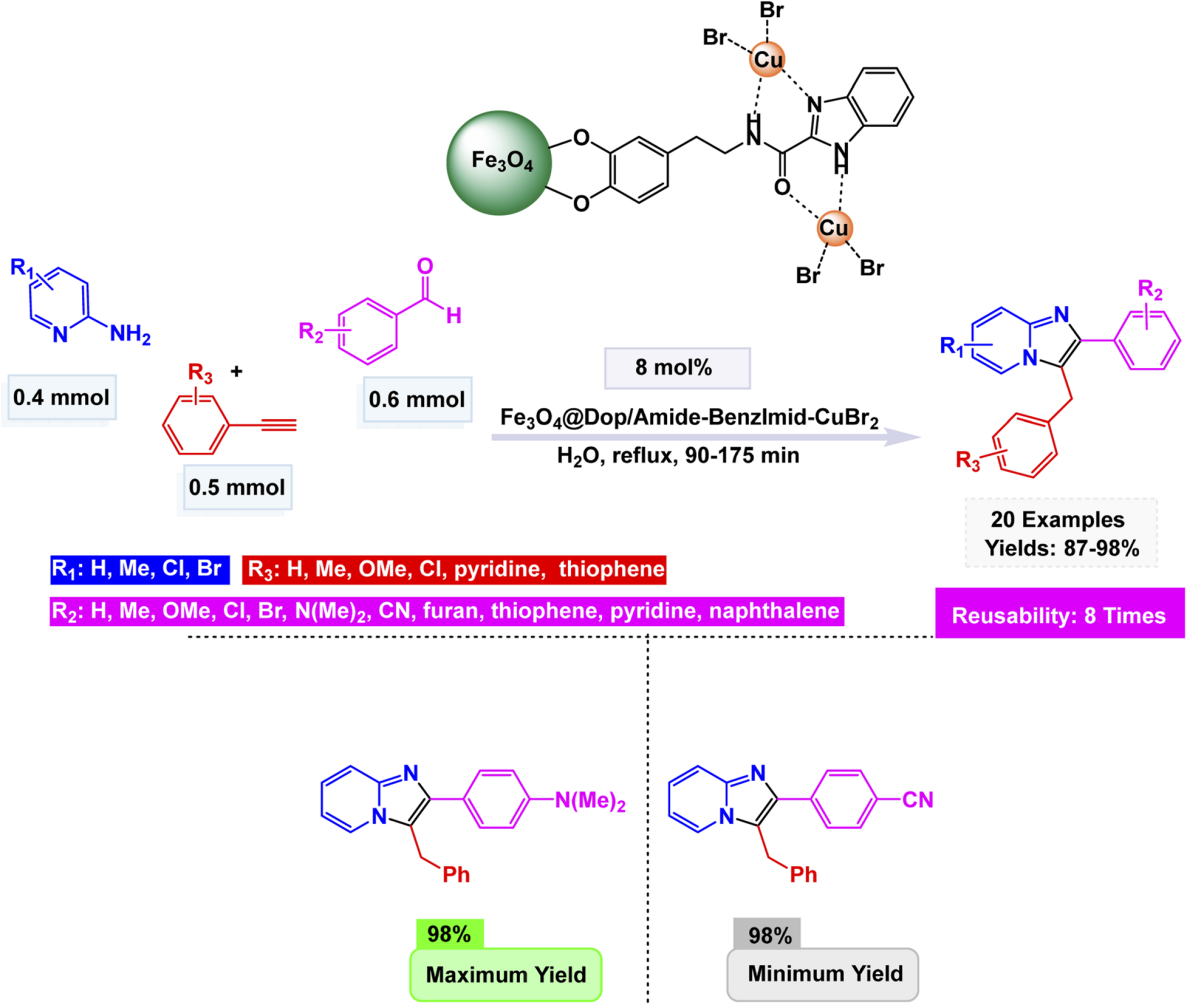
Fe_3_O_4_@Dop/Amide-BenzImid–CuBr_2_-catalyzed one-pot synthesis of imidazo[1,2-*a*]pyridines.

Yunhong Li and his team presented an innovative approach to creating a magnetically recoverable copper nanocatalyst, known as Fe_3_O_4_@Dop/Amide-BenzImid–CuBr_2_. This remarkable catalyst was developed through a meticulous process of sequential surface modification. First, they adorned Fe_3_O_4_ nanoparticles with dopamine (Dop), a compound known for its adhesive properties. Next, they introduced 1*H*-benzo[d]imidazole-2-carboxylic acid (Amide-BenzImid), further enhancing the nanoparticles' functionalization. Finally, they coordinated these modified nanoparticles with CuBr_2_, resulting in a sophisticated catalyst that offers both efficiency and the convenience of magnetic recovery in catalytic processes.^152^ Analysis presented for this catalyst, revealing spherical nanoparticles (15–30 nm) with strong magnetic properties (45.1 emu g^−1^) and thermal stability up to 700 °C. The nanocatalyst (8 mol%) exhibited remarkable activity in the one-pot three-component synthesis of imidazo[1,2-*a*]pyridines at refluxing water, affording yields up to 98% Scheme 14. The proposed mechanism involves condensation between 2-aminopyridine and the aldehyde to give an imine intermediate, followed by copper-assisted alkyne activation, nucleophilic addition, and oxidative cyclization to generate the fused heterocycle. The inclusion of electron-donating groups on aromatic rings significantly boosted product yields, creating a marked improvement in the overall output. In contrast, the presence of electron-withdrawing groups had a negligible effect, leaving the yields relatively unchanged. The Fe_3_O_4_@Dop/Amide-BenzImid–CuBr_2_ catalyst preserved its distinct shape and metal composition even after undergoing eight consecutive cycles, showcasing an impressive resilience with minimal decline in efficiency. This remarkable performance underscores its magnetic stability and robustness, demonstrating exceptional durability in environmentally friendly aqueous conditions.

**Scheme 15:**
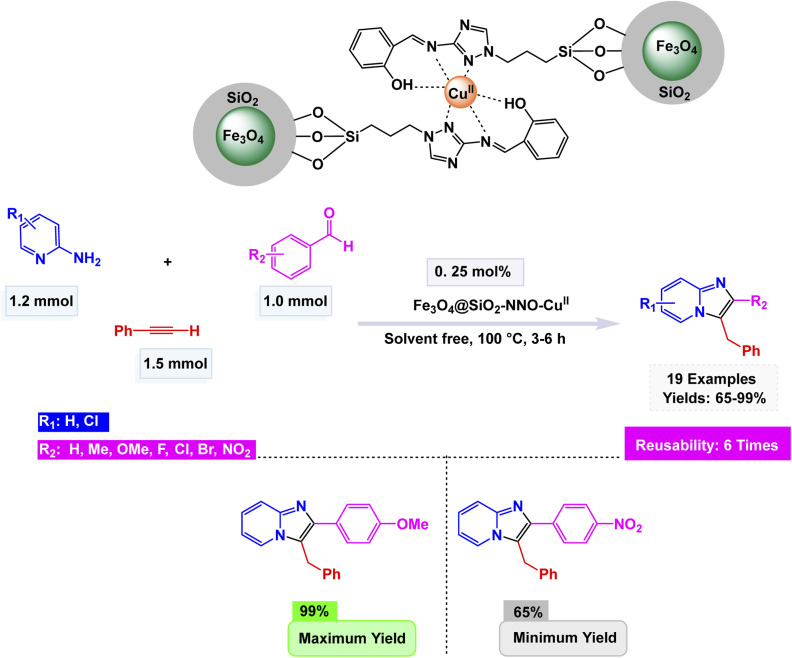
Fe_3_O_4_@SiO_2_–NNO–Cu(ii)-catalyzed synthesis of imidazo[1,2-*a*]pyridines.

Continuing this line of research, Rafiee and Hasani prepared a copper-based magnetic nanocatalyst, Fe_3_O_4_@SiO_2_–NNO–Cu(ii), through covalent immobilization of a tridentate Schiff base ligand derived from salicylaldehyde and 3-amino-1,2,4-triazole onto silica-coated Fe_3_O_4_ nanoparticles.^153^ The nanocomposite was meticulously analyzed through a battery of advanced characterization techniques, including FT-IR, EDX, SEM, XRD, ICP-OES, and VSM. These analyses confirmed a strikingly uniform spherical morphology, measuring approximately 30 nm in diameter, accompanied by a copper loading of 2.86 wt%. Its impressive saturation magnetization value of 37.71 emu g^−1^ ensured highly efficient magnetic recovery. The catalyst demonstrated exceptional catalytic prowess in the solvent-free A^3^-coupling reaction, skillfully bringing together benzaldehydes, 2-aminopyridines, and terminal alkynes to yield imidazo[1,2-*a*]pyridine derivatives with remarkable yields, as illustrated in Scheme 15. The protocol accommodated both aromatic and aliphatic aldehydes and alkynes, showing high tolerance toward electron-withdrawing and electron-donating substituents, with yields reaching up to 99%. The nanocatalyst demonstrated exceptional structural integrity and sustained catalytic efficiency over six consecutive cycles, showcasing its robustness, non-leaching behavior, and significant potential for promoting environmentally friendly heterocyclic synthesis.

**Scheme 16:**
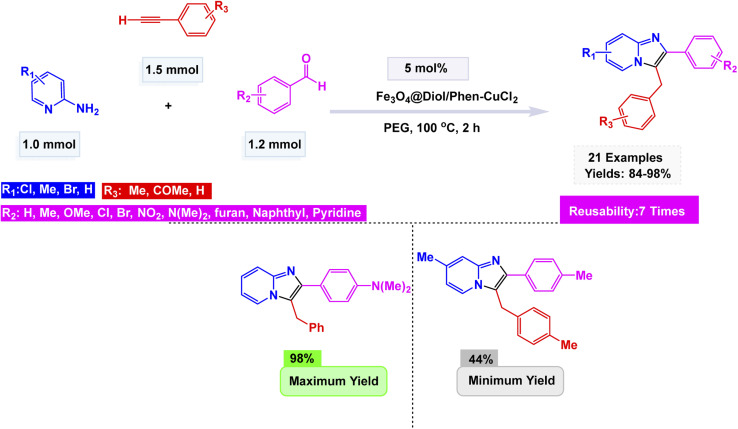
Fe_3_O_4_@Diol/Phen–CuCl_2_-catalyzed synthesis of imidazo[1,2-*a*] in PEG.

Jiang *et al.* developed a magnetically recoverable copper nanocatalyst, Fe_3_O_4_@Diol/Phen–CuCl_2_, prepared by sequential functionalization of Fe_3_O_4_ nanoparticles with 1,10-phenanthroline-5,6-diol (Diol/Phen) followed by coordination with CuCl_2_.^154^ The resulting nanocomposite was comprehensively characterized by FT-IR, XRD, SEM, TEM, EDX, TGA, VSM, and ICP-OES, revealing nearly spherical particles (15–25 nm) with a uniform core–shell morphology and strong magnetic behavior (45.18 emu g^−1^). This innovative catalyst, comprising 8 mol% of a finely-tuned nanomaterial, masterfully facilitated a one-pot, three-component coupling involving 2-aminopyridine, a variety of aromatic aldehydes, and terminal alkynes, all within a PEG solvent at a steady temperature of 100 °C. The result An impressive array of imidazo[1,2-*a*]pyridine derivatives, yielded in remarkable quantities ranging from 84% to 98% see Scheme 16. This reaction demonstrated an exceptional tolerance for a diverse range of substrates, whether adorned with electron-donating or electron-withdrawing substituents on the aryl ring, and consistently maintained its high efficiency throughout. The Fe_3_O_4_@Diol/Phen–CuCl_2_ nanocatalyst itself revealed an astonishing level of stability, showcasing its ability to be magnetically retrieved after use and reused for seven consecutive cycles, all while exhibiting no significant decline in catalytic performance or structural integrity. This was further validated by meticulous post-reaction analyses using VSM and TEM.

### 2.4. Catalysis by magnetic nanoparticles supported zinc catalyst

**Scheme 17:**
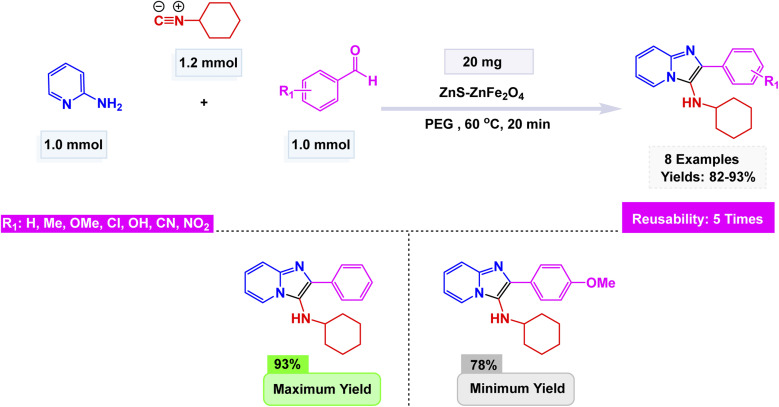
ZnS–ZnFe_2_O_4_ catalyzed synthesis of imidazo[1,2-*a*]pyridines.

**Scheme 18:**
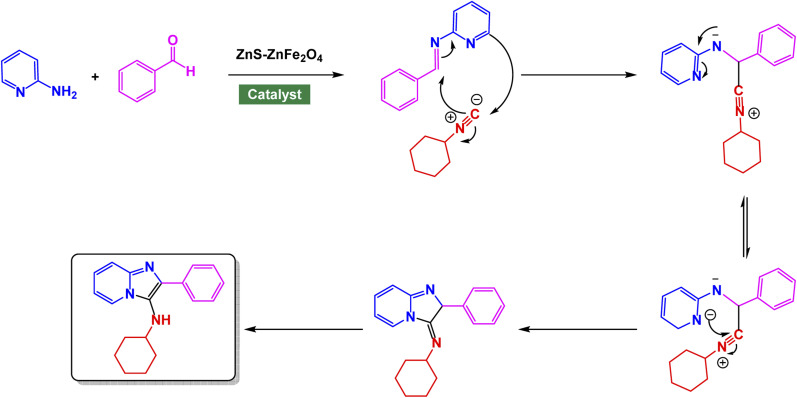
Plausible mechanism for ZnS–ZnFe_2_O_4_ catalyzed synthesis of imidazo[1,2-*a*]pyridines.

Zinc is an effective catalyst in many organic and industrial reactions because it offers mild Lewis acidity and can activate carbonyl compounds and other electrophiles without harsh conditions.^155–157^ Its low toxicity, natural abundance, and compatibility with green chemistry make zinc-based catalysts attractive alternatives to more expensive or hazardous metals.^158,159^ Magnetic nanoparticles supported with zinc catalysts offer an efficient and reusable platform for promoting a variety of organic transformations, benefiting from the high surface area and stability of the magnetic support. The magnetic core allows for quick catalyst separation using an external magnet, while the zinc species provide mild, selective catalytic activity suitable for green and sustainable synthesis. In this respect, advancing the application of ferrite-based nanocatalysts, Ichie and Garza designed a bifunctional ZnS–ZnFe_2_O_4_ nanocatalyst through a simple precipitation–sonication method using metal nitrates and thioacetamide (ZnS), producing uniform spherical nanoparticles (45 nm) with excellent thermal stability as verified by FT-IR, SEM, and TGA analyses.^104^ The catalyst efficiently promoted the Groebke–Blackburn–Bienaymé reaction of 2-aminopyridine, aromatic aldehydes, and cyclohexyl isocyanide in PEG solvent at 60 °C, affording imidazo[1,2-*a*]pyridine derivatives Scheme 17. The reaction mechanism Scheme 18 involves initial imine formation between 2-aminopyridine and aromatic aldehyde, followed by isocyanide addition, intramolecular cyclization, and tautomerization to form the fused heterocyclic product. The synergistic interaction of Zn^2+^ and Fe^3+^ centers facilitates both carbonyl activation and cyclization, leading to enhanced reactivity and selectivity. The ZnS–ZnFe_2_O_4_ catalyst exhibited exceptional magnetic recoverability and maintained its impressive activity over five consecutive cycles, emphasizing its robustness, reusability, and promising eco-friendly potential for sustainable heterocyclic synthesis.

### 2.5. Catalysis by magnetic nanoparticles supported manganese catalyst

Manganese (Mn) catalysts are valued for their earth-abundance, low toxicity, and ability to facilitate a wide range of oxidation, reduction, and C–H activation reactions.^160,161^ Their tunable oxidation states allow Mn-based systems to mimic enzymatic processes and enable environmentally friendly catalytic transformations in both organic and industrial chemistry.^130,162^ Magnetic nanoparticles supported manganese catalysts combine the redox versatility of manganese with the easy recoverability and high surface area of magnetic nanomaterials, creating efficient and reusable catalytic systems.^130^ This hybrid design enhances catalyst stability, improves reaction rates, and supports greener processes by allowing simple magnetic separation and repeated recycling without significant loss of activity.

**Scheme 19:**
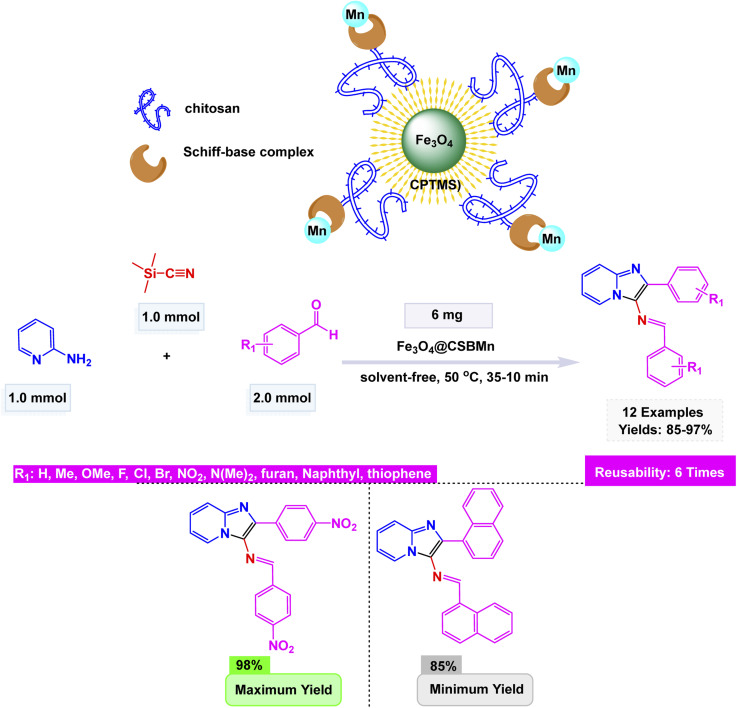
Fe_3_O_4_@CSBMn catalyzed synthesis of imidazo[1,2-*a*]pyridines.

Through an innovative strategy, Rakhtshah and Yaghoobi engineered a remarkable magnetically recoverable nanocatalyst, named Fe_3_O_4_@CSBMn. This unique creation features a manganese Schiff-base complex (BMn) intricately immobilized on a foundation of chitosan (CS)-coated Fe_3_O_4_ nanoparticles, combining the strengths of magnetism and biocompatibility to achieve exceptional catalytic performance.^163^ The catalyst was synthesized through sequential functionalization steps involving chitosan coating, Schiff-base formation with 2-formylpyridine, and Mn(ii) coordination, and was fully characterized by FT-IR, XRD, TEM, EDX, VSM, and TGA, confirming a stable core–shell structure with a particle size of about 30 nm and superparamagnetic behavior (Ms = 27.03 emu g^−1^). The innovative nanocatalyst remarkably facilitated the efficient one-pot, solvent-free synthesis of 3-iminoaryl-imidazo[1,2-*a*]pyridines. This transformation unfolded through the elegant condensation of 2-aminopyridine, aryl aldehydes, and TMSCN at a moderate temperature of 50 °C. The reaction yielded the desired products in impressive yields ranging from 85% to 97%, all within a swift timeframe of 10 to 35 minutes as illustrated in Scheme 19. This fascinating process is driven by the strategic activation of the aldehyde using the Lewis-acidic Mn(ii) centre. Subsequently, the cyanide moiety is introduced, leading to an intricate intramolecular cyclization that forms the final imidazo[1,2-a]pyridine structures. This transformation not only showcases the catalytic prowess of the nanomaterial but also highlights the elegant interplay of reaction components in achieving such high yields efficiently. The Fe_3_O_4_@CSBMn catalyst was magnetically separable and reused for six consecutive runs with negligible loss of activity or change in structure, highlighting its stability, efficiency, and environmental compatibility in the sustainable synthesis of imidazo[1,2-a]pyridines.

### 2.6. Catalysis by magnetic nanoparticles supported antimony catalyst

Antimony (Sb) catalysts are known for their strong Lewis acidity, which makes them useful in promoting reactions like polymerizations, esterifications, and various carbon–heteroatom bond-forming processes.^164^ Because Sb species can be supported on solid materials or used in heterogeneous form, they often provide good activity while remaining easy to recover and reuse.^165^ Magnetic nanoparticles supported antimony catalysts integrate the catalytic activity of antimony with the convenient magnetic recoverability of nanoparticle supports, enabling efficient, stable, and easily recyclable catalytic systems.

**Scheme 20:**
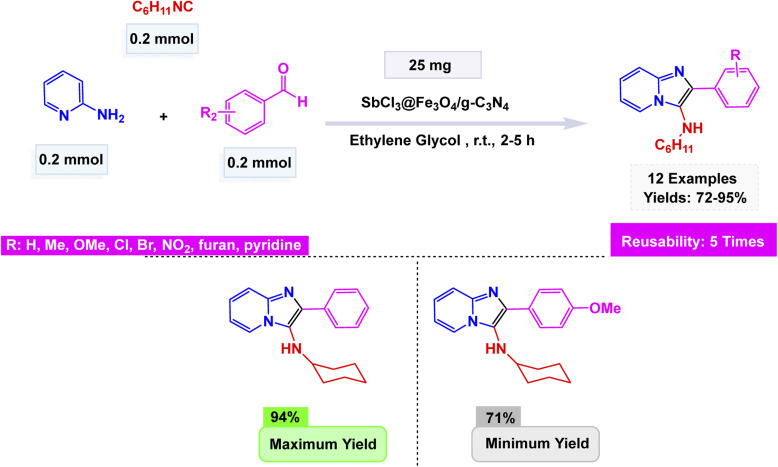
SbCl_3_@Fe_3_O_4_/g-C_3_N_4_ catalyzed synthesis of imidazo[1,2-*a*]pyridines.

Extending the sequence of green catalytic strategies, Azhdari and co-workers developed an efficient SbCl_3_@Fe_3_O_4_/g-C_3_N_4_ nanocomposite through a straightforward solvent-evaporation approach that integrates magnetic functionality with Lewis acidity.^166^ The composite was constructed by co-immobilizing Fe_3_O_4_ nanoparticles and Sb(iii) species onto exfoliated graphitic carbon nitride nanosheets, forming a stable, magnetically responsive hybrid with uniformly dispersed active sites. Structural analyses (FT-IR, XRD, SEM, and EDX) confirmed the chemical integrity of the g-C_3_N_4_ matrix and successful loading of both Fe_3_O_4_ and SbCl_3_. The catalyst exhibited outstanding performance in the Groebke–Blackburn–Bienaymé multicomponent reaction of 2-aminopyridine, aromatic aldehydes, and isocyanides under room-temperature conditions in ethylene glycol, affording 3-aminoimidazo[1,2-*a*]pyridine derivatives in yields up to 95% within 2–5 h Scheme 20. The reaction mechanism proceeds through imine formation followed by isocyanide insertion and cyclization, facilitated by the cooperative action of Sb(iii) Lewis-acid sites and the magnetic g-C_3_N_4_ framework, which enhances both substrate activation and electron transfer. Moreover, the catalyst retained high activity for at least five cycles without loss of efficiency.

### 2.7. Catalysis by magnetic nanoparticles supported ionic liquids

Ionic liquids stand out as remarkably effective catalysts, primarily due to their distinctive attributes such as tunable acidity and basicity, unparalleled thermal stability, and their ability to solubilize a diverse range of both organic and inorganic compounds.^167,168^ Their ionic character fosters an exceptionally organized liquid milieu that can significantly amplify reaction rates and improve selectivity. Moreover, their negligible vapor pressure positions them as environmentally friendly alternatives to traditional volatile organic solvents.^169^ Perhaps most compelling is their potential for recyclability; ionic liquids can often be reused with minimal degradation in catalytic activity, rendering them highly appealing for eco-conscious and sustainable catalytic processes. When combined with magnetic nanoparticles, ionic liquids take on a new dimension, merging the high efficiency and adaptability of ionic-liquid catalysts with the effortless recovery and recyclability offered by magnetically-responsive nanomaterials.^170^ This synergy not only enhances reaction performance but also aligns seamlessly with the principles of green chemistry.

**Scheme 21:**
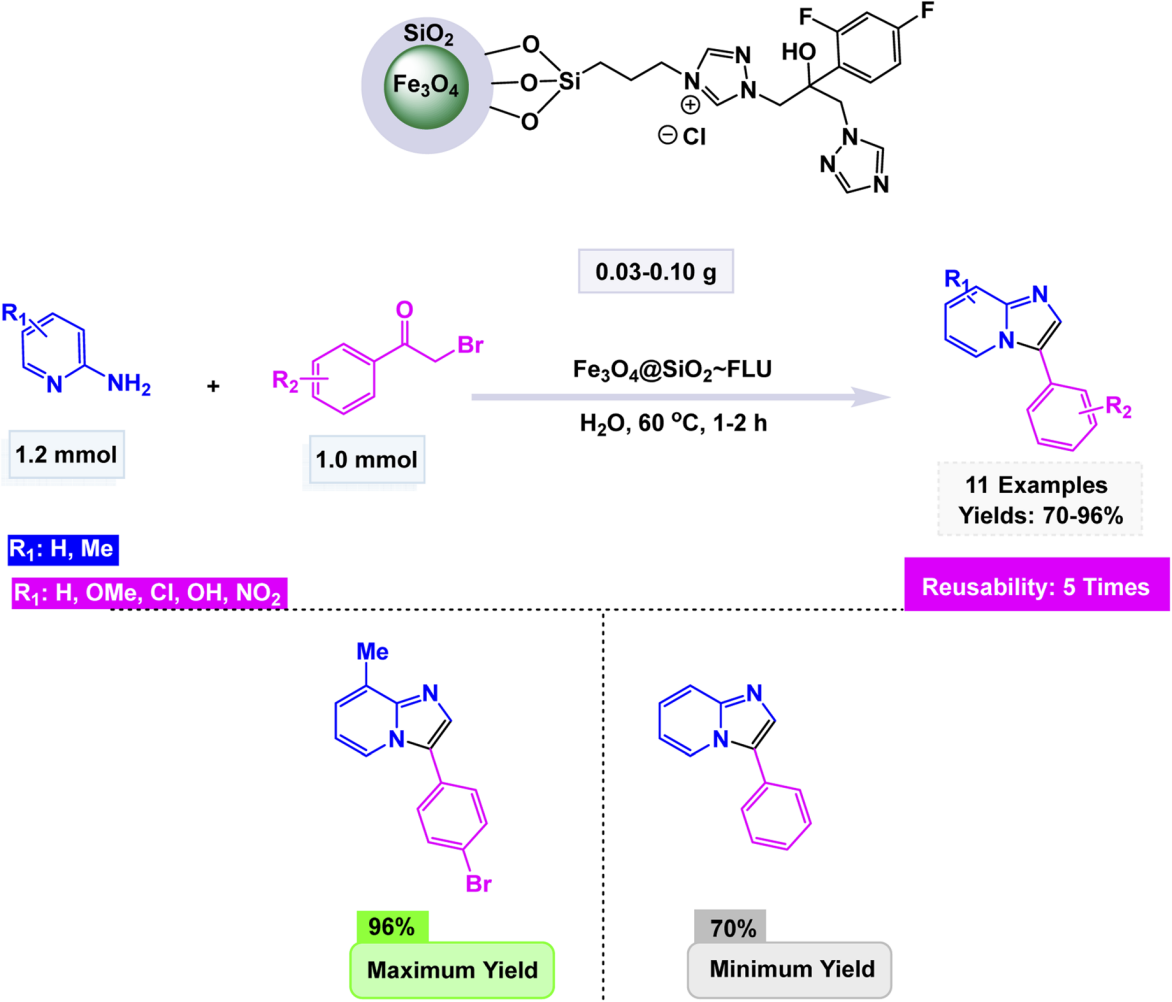
Fe_3_O_4_@SiO_2_–FLU catalyzed synthesis of 3-aryl-imidazo[1,2-*a*]pyridines.

Using this strategy, Jafarzadeh and co-workers introduced a magnetically recoverable fluconazole-functionalized Fe_3_O_4_@SiO_2_ nanocatalyst (Fe_3_O_4_@SiO_2_–FLU) for the green synthesis of 3-aryl- and 3-amino-imidazo[1,2-*a*]pyridines.^171^ The catalyst was prepared by immobilizing fluconazole onto chloropropyl-modified silica-coated magnetite through a nucleophilic substitution process, and its formation was confirmed by FT-IR, XRD, TEM, SEM, EDX, TGA, and CHN analyses, showing a core–shell morphology with an average size of about 40–45 nm and a crystalline spinel structure. The Fe_3_O_4_@SiO_2_–FLU nanocatalyst showcased remarkable efficiency in facilitating the condensation reaction between phenacyl bromides and 2-aminopyridine in an aqueous environment, resulting in the formation of 3-aryl-imidazo[1,2-*a*]pyridines with impressive yields see Scheme 21. Additionally, this versatile catalyst excelled in orchestrating a three-component coupling reaction involving benzaldehydes, 2-aminopyridine, and isocyanides, all under solvent-free conditions, culminating in the synthesis of 3-amino-imidazo[1,2-*a*]pyridines. The underlying mechanism is characterized by the nucleophilic substitution of bromide by the amino group, which is then followed by a seamless intramolecular cyclization and subsequent dehydration process, weaving together the intricate tapestry of these complex molecular structures. The Fe_3_O_4_@SiO_2_ – FLU catalyst demonstrated excellent tolerance to various substituents, achieved higher conversions with electron-withdrawing groups, and remained magnetically separable and stable through 5 cycles of reuse under mild operating conditions, highlighting its environmentally benign nature.

**Scheme 22:**
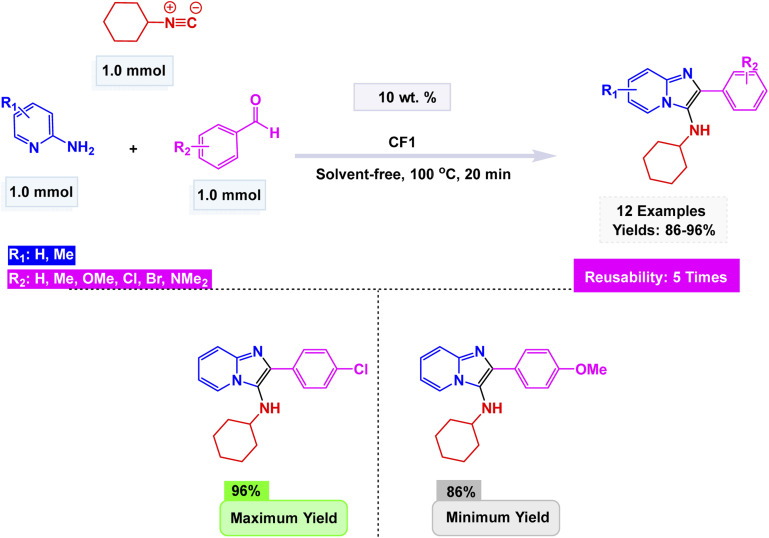
CoFe_2_O_4_-ionic liquid catalyzed synthesis of 3-aminoimidazo[1,2-*a*]pyridines.

In a further development of magnetic nanocatalyst-based strategies, Tavakkoli *et al.* developed a mesoporous cobalt ferrite (CoFe_2_O_4_) catalyst *via* an ionic liquid-assisted hydrothermal route using octyl-4-aza-1-azoniabicyclo[2.2.2]octane bromide as a morphology-directing template.^172^ The ionic liquid played a decisive role in tuning the nanostructure, leading to well-defined cubic spinel phases with diverse morphologies—ranging from nanoparticles to nanorods and nanosheets—while enhancing surface area and magnetic responsiveness. The optimized CoFe_2_O_4_ (CF1) sample exhibited excellent catalytic activity in the Groebke–Blackburn–Bienaymé (GBB) multicomponent reaction involving 2-aminopyridine, benzaldehyde, and isocyanide under solvent-free conditions at 100 °C, affording 3-aminoimidazo[1,2-*a*]pyridine derivatives in high yields within 20 min Scheme 22. Mechanistically, the process involves initial imine formation between 2-aminopyridine and aldehyde, followed by isocyanide addition and intramolecular cyclization to generate the fused heterocycle. The Lewis-acidic Co^2+^ and Fe^3+^ centers facilitate substrate polarization and activation throughout the catalytic cycle. Furthermore, the catalyst demonstrated exceptional durability, maintaining its performance across five consecutive cycles with no discernible decline in activity.

**Scheme 23:**
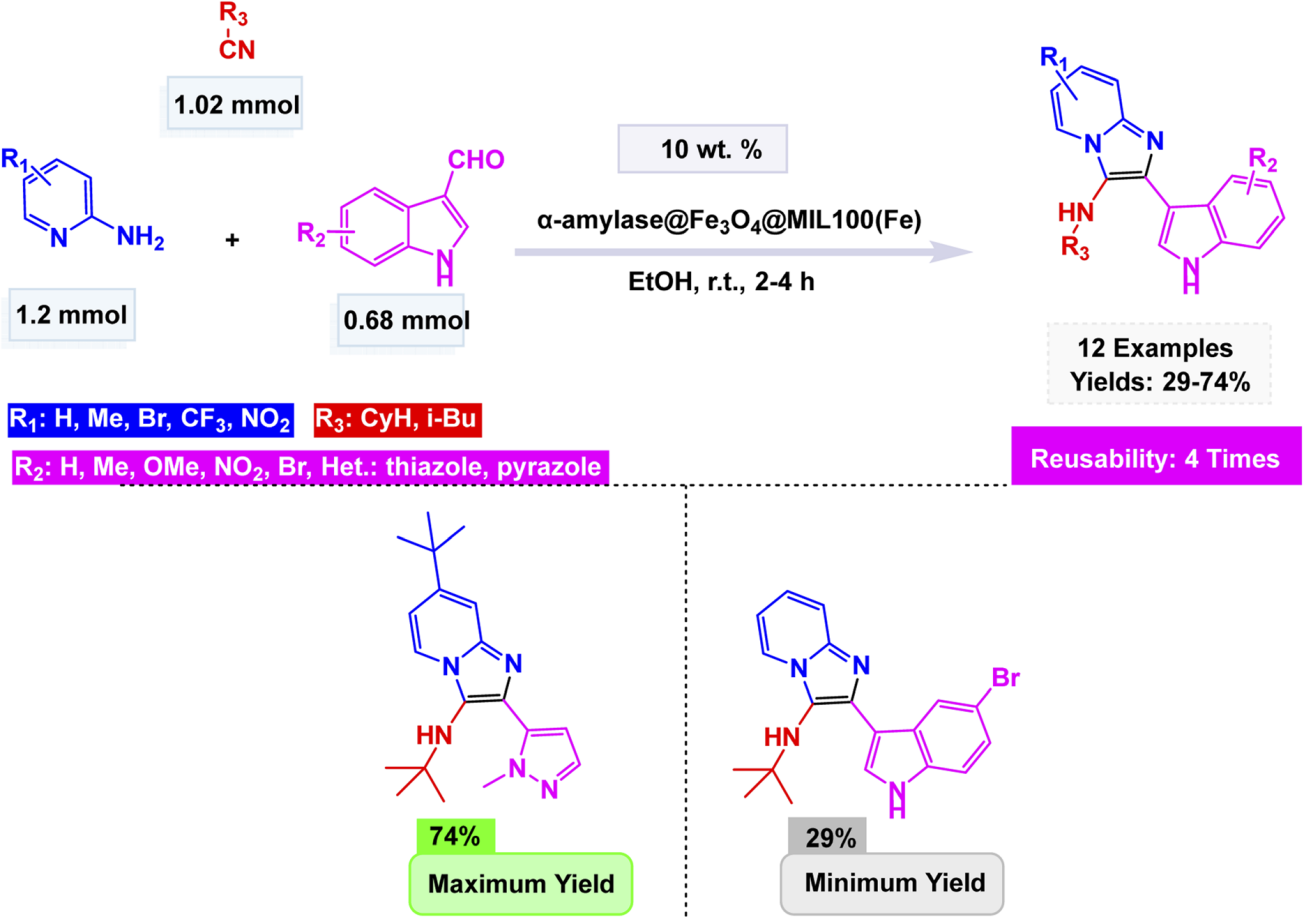
α-Amylase@Fe_3_O_4_@MIL-100(Fe)-catalyzed synthesis of indole-based imidazo[1,2-*a*]pyridines.

Broadening the horizon of eco-friendly catalytic systems, Kamboj and Tyagi developed a biocatalytic route employing α-amylase for the sustainable synthesis of indole-based imidazo[1,2-*a*]pyridines.^173^ The transformation proceeded *via* a Groebke–Blackburn–Bienaymé multicomponent reaction, in which 2-aminopyridine, indole-3-carboxaldehyde, and *tert*-butyl isocyanide reacted efficiently under mild, solvent-free or ethanolic conditions to afford the target heterocycles in excellent yields Scheme 23. Mechanistically, the reaction begins with enzyme-assisted imine formation between 2-aminopyridine and indole-3-carboxaldehyde, followed by isocyanide incorporation and a [4 + 1] cycloaddition step, ultimately leading to the fused imidazopyridine scaffold through proton rearrangement. For improved recyclability and operational simplicity, α-amylase was immobilized on Fe_3_O_4_@MIL-100(Fe), producing a magnetically recoverable hybrid biocatalyst. The immobilized enzyme retained its activity and selectivity over four consecutive reaction cycles without notable deactivation. This innovative combination of biocatalysis and magnetic nanotechnology demonstrates an efficient, reusable, and environmentally benign approach for constructing nitrogen-fused heterocycles.

**Scheme 24:**
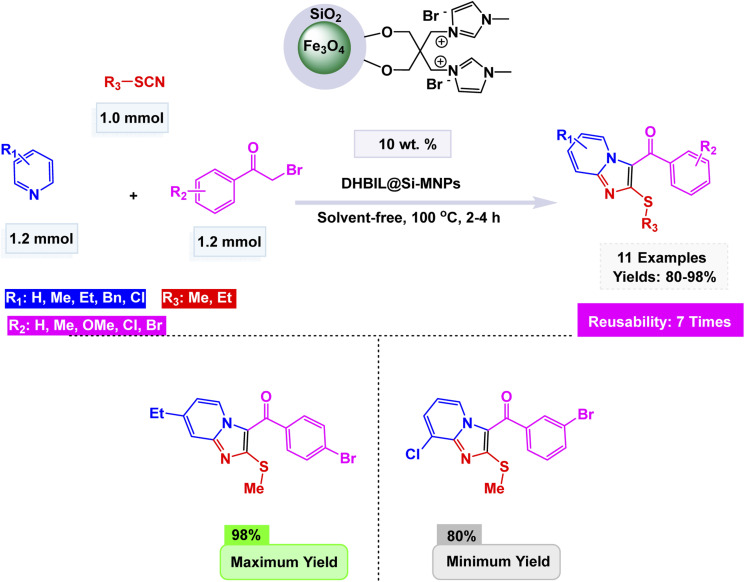
DHBIL@Si–MNPs-catalyzed synthesis of imidazo[1,2-*a*]pyridines.

**Scheme 25:**
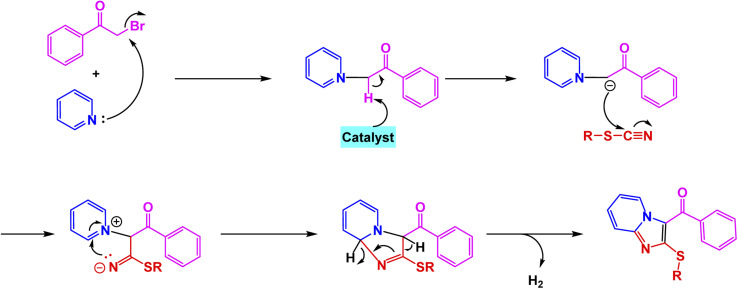
Proposed mechanism for the synthesis of imidazo[1,2-*a*]pyridine derivatives.

Extending the trend of developing efficient magnetic nanocatalysts, Salimi and co-workers developed a basic ionic liquid-functionalized magnetic silica nanocatalyst (DHBIL@Si – MNPs) for the sustainable synthesis of imidazo[1,2-*a*]pyridines.^174^ The catalyst was prepared by immobilizing a dihydroxyl-based basic ionic liquid onto Fe_3_O_4_–SiO_2_ nanoparticles, forming a thermally stable, magnetically separable system with strong basicity and high surface area. Characterization by FT-IR, XRD, SEM, TEM, TGA, and VSM confirmed the core–shell structure and superparamagnetic properties. The catalyst effectively promoted the three-component reaction of pyridines, phenacyl bromides, and thiocyanates, affording imidazo[1,2-*a*]pyridine derivatives in excellent yields Scheme 24. The mechanistic pathway Scheme 25 involves an initial nucleophilic substitution of bromide by pyridine, followed by attack of thiocyanate and intramolecular cyclization to form the fused heterocyclic framework. The catalyst was magnetically recoverable and reused for 7 cycles without notable loss of activity.

### 2.8. Catalysis by magnetic nanoparticles supported acidic catalysts

Acids are widely used as catalysts because they can donate protons to activate reactants, thereby accelerating a broad range of chemical transformations such as esterification, hydrolysis, dehydration, and polymerization.^175^ By increasing the electrophilicity of functional groups or stabilizing reaction intermediates, acid catalysts help lower activation energies and improve reaction rates and selectivity.^176^ They can be employed in either homogeneous form—such as mineral acids like sulfuric or hydrochloric acid—or heterogeneous form, including solid acids like zeolites, sulfonated polymers, or metal oxides, which offer advantages in separation and reuse.^177^ In industrial processes, acid catalysis is essential due to its cost-effectiveness, versatility, and ability to drive reactions under milder conditions, making it a cornerstone of both organic synthesis and large-scale chemical manufacturing. Magnetic nanoparticles supported acidic catalysts combine strong acid sites with magnetically responsive supports, providing high catalytic activity along with rapid and simple recovery.^178^ Their robust structure and reusability make them attractive for green and scalable chemical processes, especially in esterification, hydrolysis, and biomass conversion reactions.

**Scheme 26:**
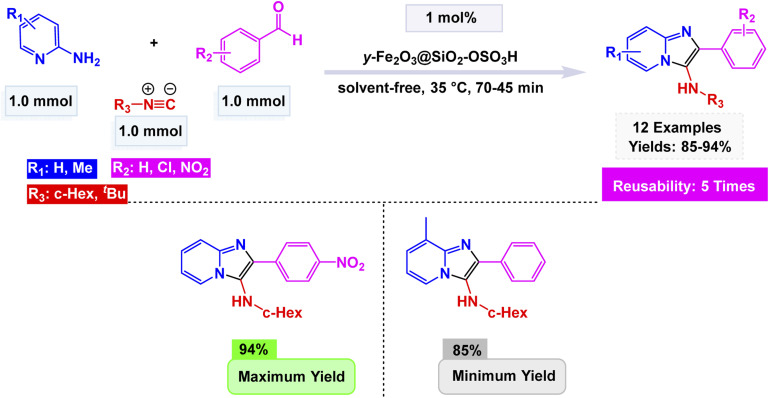
γ-Fe_2_O_3_@SiO_2_–OSO_3_H catalyzed Ugi-like Groebke–Blackburn–Bienaymé synthesis of 3-aminoimidazo[1,2-*a*]pyridines.

**Scheme 27:**
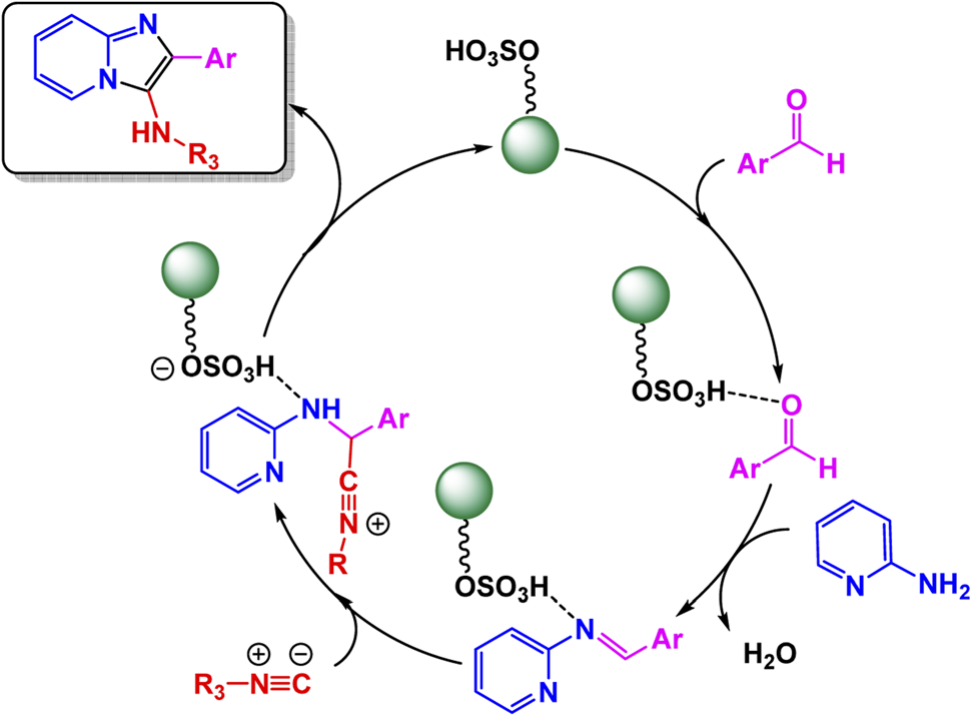
Plausible mechanism for the γ-Fe_2_O_3_@SiO_2_–OSO_3_H catalyzed formation of 3-aminoimidazo[1,2-*a*]pyridines.

Similarly, Rostamnia *et al.* prepared γ-Fe_2_O_3_@SiO_2_–OSO_3_H by coating maghemite nanoparticles with silica followed by sulfonation using chlorosulfonic acid, and characterized the material by XRD, SEM, and FT-IR analyses, confirming successful acid functionalization.^179^ A catalytic amount (1 mol%) of this nanomagnetically modified sulfuric acid was used for the Ugi-like Groebke–Blackburn–Bienaymé (GBB) under solvent-free conditions, affording 3-aminoimidazo[1,2-*a*]pyridine derivatives in high yields up to 94% Scheme 26. The proposed mechanism Scheme 27 involves initial imine formation followed by isocyanide addition and cyclization through intramolecular nucleophilic attack, generating the fused heterocyclic system. Additionally, the same catalyst efficiently promoted the synthesis of aminoimidazothiazoles *via* analogous Ugi-type condensation of 2-aminothiazole, aldehydes, and isocyanides, providing up to 87% yield under identical conditions. The γ-Fe_2_O_3_@SiO_2_–OSO_3_H nanocatalyst exhibited excellent magnetic recoverability and was reused for five consecutive cycles without noticeable loss of activity, demonstrating its robustness and green applicability in multicomponent heterocyclic synthesis.

**Scheme 28:**
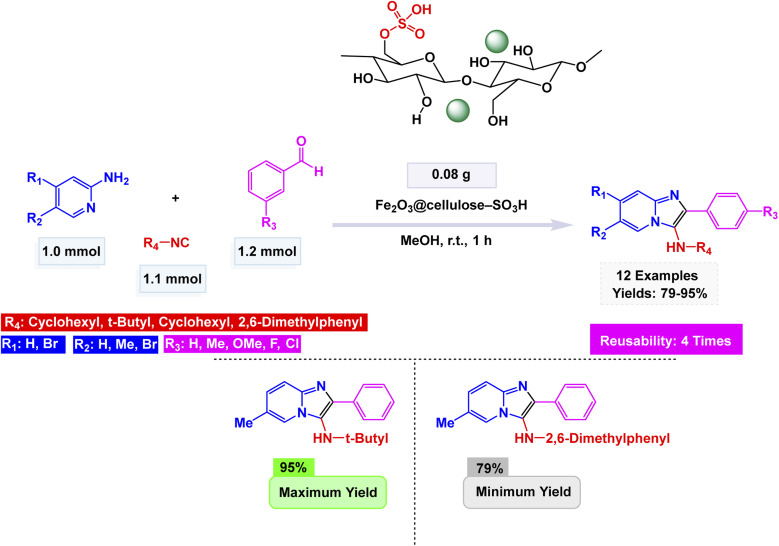
Fe_2_O_3_@cellulose–SO_3_H nanocomposite catalyzed synthesis of 3-aminoimidazo[1,2-*a*]pyridines.

Shaabani *et al.* synthesized Fe_2_O_3_@cellulose and subsequently sulfonated it with chlorosulfonic acid in CHCl_3_ at 0 °C to obtain Fe_2_O_3_@cellulose–SO_3_H. Both nanocomposites were characterized by FAAS, XRD, and SEM, showing Fe_2_O_3_ loadings of about 23% and 21%, respectively, with preserved cellulose morphology. Catalytic quantities of 0.08 g for Fe_2_O_3_@cellulose–SO_3_H and 0.07 g for Fe_2_O_3_@cellulose were employed.^180^ These bio-supported nanocatalysts were applied in the oxidative Groebke–Blackburn–Bienaymé (OGBB-3CR) of alcohols, 2-aminopyridines, and isocyanides to produce 3-aminoimidazo[1,2-*a*]pyridines Scheme 28, as well as in two related reactions: the classical GBB using aldehydes and the oxidative variant using Fe_2_O_3_@cellulose. Both electron-donating and electron-withdrawing substituents on the aryl moieties were well tolerated, giving high to excellent yields across substrates. The catalysts demonstrated remarkable magnetically recoverable and reusable properties, maintaining their efficiency across four consecutive cycles without any significant reduction in efficiency.

**Scheme 29:**
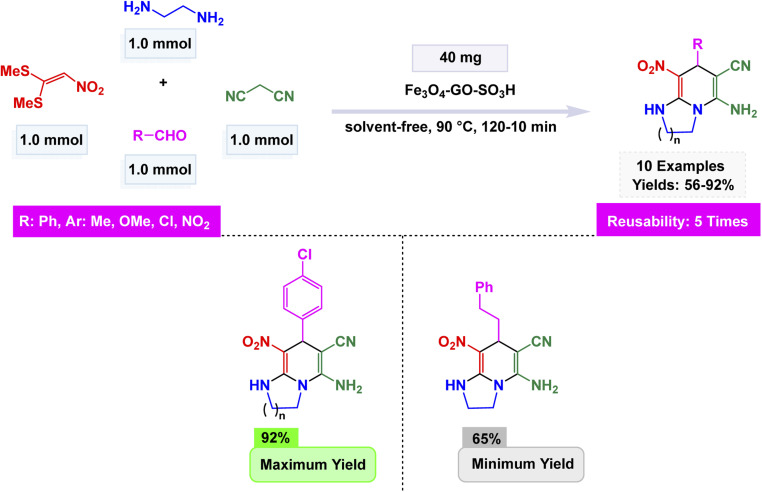
Fe_3_O_4_–GO–SO_3_H catalyzed synthesis of aryl-substituted imidazo[1,2-*a*]pyridines.

**Scheme 30:**
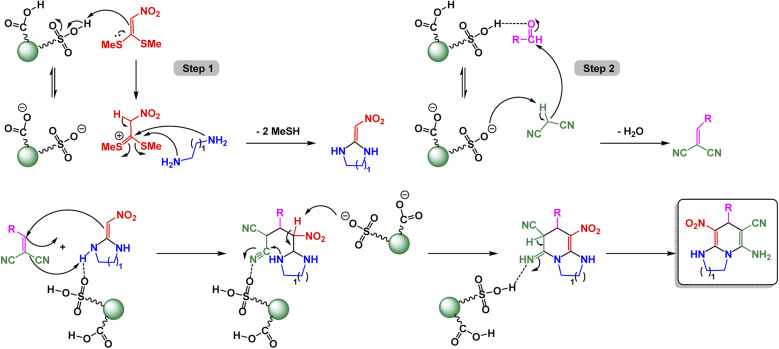
Proposed mechanism for the Fe_3_O_4_–GO–SO_3_H catalyzed synthesis of aryl-substituted imidazo[1,2-*a*]pyridines.

Karimiyan and Rostamizadeh prepared Fe_3_O_4_–GO–SO_3_H by sulfonating Fe_3_O_4_–graphene oxide with chlorosulfonic acid in dichloromethane and confirmed its structure using TEM, TGA, and EDX analyses.^181^ A catalyst amount of 40 mg was applied to synthesize aryl-substituted imidazo[1,2-*a*]pyridines under solvent-free conditions Scheme 29. The reaction tolerated both electron-donating and electron-withdrawing groups, providing yields of 65–92%. The mechanism Scheme 30 proceeds through the formation of a heterocyclic ketene aminal from the nitroethylene and diamine, followed by Knoevenagel condensation of the aldehyde with malononitrile, and an aza-ene addition between the two intermediates. Subsequent imine–enamine tautomerization, intramolecular N-cyclization, and a 1,3-hydride shift lead to the fused heterocyclic product. Additionally, the same Fe_3_O_4_–GO–SO_3_H catalyst efficiently mediated analogous multicomponent reactions when isatin derivatives replaced aldehydes, yielding spiro-oxindolo-imidazo[1,2-*a*]pyridines and pyrido[1,2-*a*]pyrimidines, or when ethyl cyanoacetate substituted malononitrile, giving spiro-oxindoles selectively *via* activation of the ester carbonyl. The catalyst exhibited excellent stability and magnetic recoverability, maintaining its activity for five consecutive cycles without measurable deactivation.

**Scheme 31:**
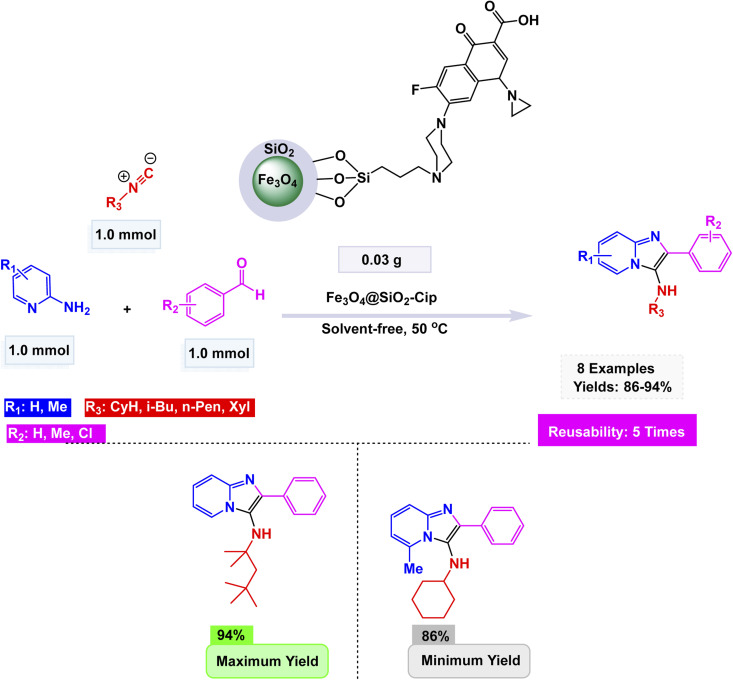
Fe_3_O_4_@SiO_2_–Cip-catalyzed synthesis of imidazo[1,2-*a*]pyridines.

**Scheme 32:**
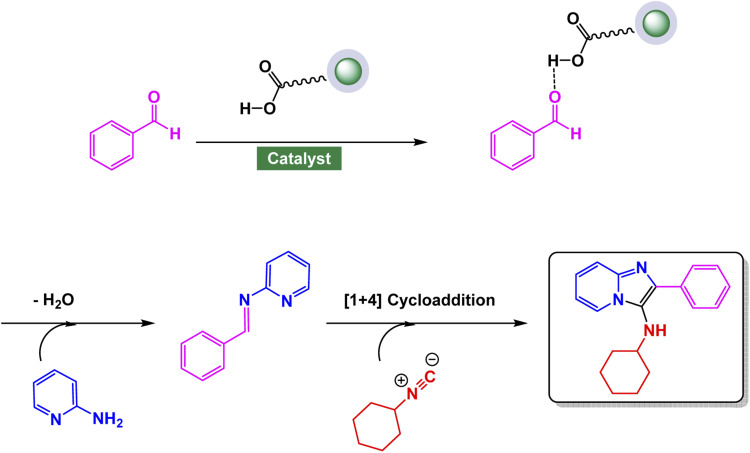
Proposed mechanism for Fe_3_O_4_@SiO_2_–Cip-catalyzed synthesis of 3-aminoimidazo[1,2-*a*]pyridines.

Soleimani and co-workers developed a ciprofloxacin-functionalized magnetic silica nanocatalyst (Fe_3_O_4_@SiO_2_–Cip) that exhibited high catalytic activity and recyclability in multicomponent heterocyclic synthesis.^174^ The catalyst was prepared through sol–gel coating of Fe_3_O_4_, surface modification with chloropropyltriethoxysilane, and covalent immobilization of ciprofloxacin. Characterization by FT-IR, XRD, TEM, SEM, EDX, TGA, and CHN confirmed its core–shell morphology and chemical stability. The catalyst efficiently promoted isocyanide-based multicomponent reactions to yield 1*H*-chromeno[2,3-d]pyrimidine-5-carboxamides and 3-aminoimidazo[1,2-*a*]pyridines under mild ethanol/water conditions Scheme 31. The reaction proceeds through imine formation between the aldehyde and amine, followed by isocyanide addition, cyclization, and tautomerization to furnish the heterocyclic scaffold Scheme 32. The ciprofloxacin moiety acts synergistically with Fe_3_O_4_ to facilitate substrate activation and proton transfer, enhancing selectivity and catalytic turnover. The Fe_3_O_4_@SiO_2_–Cip nanocatalyst showcased remarkable versatility, effortlessly harnessed through magnetic recovery. It demonstrated impressive endurance, maintaining its efficiency across five consecutive applications, each run showcasing its unwavering performance and reliability.

### 2.9. Catalysis by magnetic nanoparticles supported organocatalysts

Organic catalysts—often called organocatalysts—are small, carbon-based molecules that accelerate chemical reactions without being consumed, typically by forming temporary, reversible interactions with reactants. These catalysts can activate molecules through mechanisms such as hydrogen bonding, proton transfer, nucleophilic catalysis, or the formation of reactive intermediates like enamines and iminium ions. Because they do not rely on metals, organic catalysts are generally more environmentally benign, less toxic, and easier to handle than many traditional inorganic catalysts. They also offer excellent functional-group tolerance and can be finely tuned through structural modifications, making them powerful tools in asymmetric synthesis, green chemistry, and modern organic transformations. Magnetic nanoparticles supported organocatalysts combine the selectivity and efficiency of organic catalysts with the easy magnetic recoverability of nanoparticle supports.^182^ This hybrid design enhances catalyst stability, enables repeated reuse, and aligns well with green chemistry principles.

**Scheme 33:**
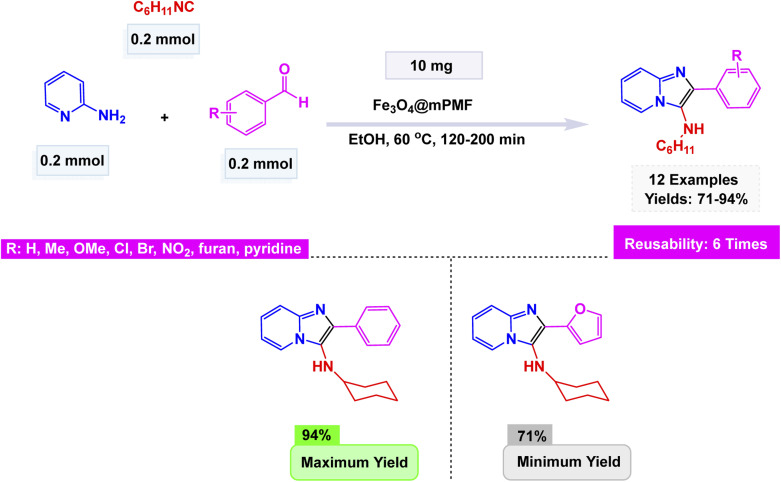
Fe_3_O_4_@mPMF-catalyzed synthesis of 3-aminoimidazo[1,2-*a*]pyridines.

In this respect, Azizi and co-workers designed a magnetic mesoporous poly-melamine–formaldehyde nanocatalyst (Fe_3_O_4_@mPMF) through a hydrothermal condensation of melamine and paraformaldehyde in the presence of Fe_3_O_4_ nanoparticles.^183^ The resulting composite exhibited a foam-like porous morphology with triazine-rich functional groups, confirmed by FT-IR, XRD, SEM, and EDS analyses. This hybrid nanostructure efficiently catalyzed the Groebke–Blackburn–Bienaymé (GBB) multicomponent reaction between 2-aminopyridine, aromatic aldehydes, and isocyanides, producing 3-aminoimidazo[1,2-*a*]pyridine derivatives in high to excellent yields Scheme 33. Mechanistically, the transformation proceeds through imine formation, followed by isocyanide addition and intramolecular cyclization, promoted by the amine-rich polymeric framework that enhances substrate activation. The catalyst demonstrated excellent magnetic separability and was reused for five successive cycles without measurable iron leaching or activity loss. This study highlights the synergy of organic–inorganic hybrid design in developing stable, green, and recyclable nanocatalysts for heterocyclic synthesis.

**Scheme 34:**
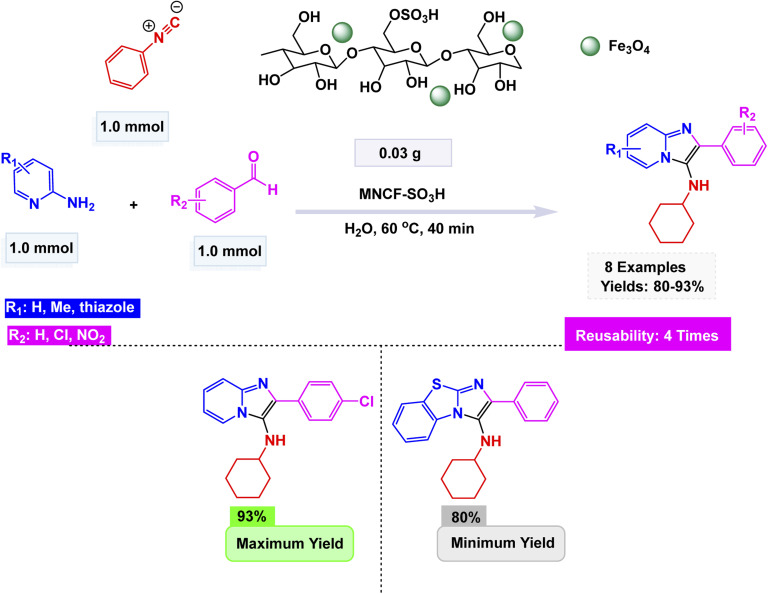
MNCF–SO_3_H-catalyzed synthesis 3-aminoimidazo[1,2-*a*]pyridines.

In continuation of the previous studies, Al-Khateeb and Nazari introduced a novel sulfonated magnetic natural cellulose fiber catalyst (MNCF–SO_3_H) designed for sustainable and recyclable organic synthesis.^184^ The catalyst was fabricated *via* sequential magnetization of sugarcane-derived cellulose fibers followed by sulfonation with chlorosulfonic acid, yielding a robust biopolymeric composite containing approximately 47% Fe_3_O_4_ and 7% –SO_3_H functionalities. Structural analysis by FT-IR, XRD, SEM, TEM, and TGA confirmed the successful incorporation of Fe_3_O_4_ nanoparticles and sulfonic acid groups within the fibrous cellulose matrix, maintaining its mesoporous structure and magnetic properties. The MNCF–SO_3_H catalyst exhibited excellent performance in two distinct multicomponent reactions: the solvent-free synthesis of bis(indolyl)methanes and the aqueous synthesis of 3-aminoimidazo[1,2-*a*]pyridines Scheme 34. Both reactions proceeded efficiently under mild and green conditions, demonstrating wide substrate tolerance toward electron-donating and electron-withdrawing groups. The superior catalytic activity was attributed to the synergistic acid-magnetic functionalities, which enhanced substrate activation and recyclability. The MNCF–SO_3_H catalyst was efficiently recovered using magnetic separation and successfully reused for four consecutive cycles, demonstrating no significant decline in performance.

## 3. Summary and outlook

With the advent of magnetic nanocatalysts, the synthesis of imidazo[1,2-*a*]pyridine derivatives has advanced significantly, bridging classical heterogeneous catalysis with modern nanotechnology-based approaches. Studies on Fe_3_O_4_, CoFe_2_O_4_, γ-Fe_2_O_3_, and functionalized hybrids demonstrate high catalytic activity driven by large surface areas and tunable surface chemistry, enabling diverse pathways such as Groebke–Blackburn–Bienaymé multicomponent reactions, A^3^-couplings, oxidative cyclizations, and classical condensations. Despite this progress, most systems have been examined with moderately substituted substrates, whereas highly substituted frameworks—particularly imidazolidine-fused analogues—remain insufficiently explored. Catalytic behavior also varies with the intrinsic nature of each nanomaterial: Lewis-acidic surfaces tend to promote imine-based activation, while basic or redox-active sites favor alkyne deprotonation or electron-transfer steps, resulting in distinct cyclization routes. From a sustainability perspective, magnetic nanocatalysts often deliver lower e-factors, improved atom economy, and reduced solvent use due to facile magnetic recovery, offering clear environmental advantages over conventional homogeneous systems. Collectively, these features position magnetic nanocatalysts as efficient, scalable, and environmentally compatible platforms for constructing imidazo[1,2-*a*]pyridine architectures.

### 3.1. Advantages of magnetic nanocatalysts compared with conventional catalysts

#### 3.1.1. Superior recovery and reusability

Magnetic separation eliminates filtration and centrifugation, unlike conventional catalysts that require time-consuming and solvent-intensive recovery steps.

High recyclability (often 5–10 cycles) with minimal activity loss, whereas many conventional catalysts deactivate rapidly due to leaching, sintering, or poisoning.

#### 3.1.2. Higher surface area and active site exposure

Magnetic nanocatalysts possess a very high surface area-to-volume ratio, exposing more catalytic sites compared with bulk catalysts.

Conventional catalysts often have limited surface accessibility, reducing catalytic efficiency and turnover frequency.

#### 3.1.3. Enhanced stability and reduced leaching

Surface coatings (SiO_2_, polymers, carbon, ligands, ionic liquids) prevent metal leaching and aggregation.

Conventional catalysts frequently suffer from metal loss, sintering, and structural collapse under reaction conditions.

#### 3.1.4. Tunable surface chemistry and improved selectivity

The surface of magnetic nanoparticles can be chemically modified to control Lewis/Brønsted acidity, basicity, redox behavior, or metal coordination.

Conventional catalysts usually have fixed surface properties with limited tunability, leading to lower selectivity in multicomponent or cyclization reactions.

#### 3.1.5. Compatibility with green and mild reaction conditions

Magnetic nanocatalysts efficiently catalyze reactions in water, PEG, ethanol, or solvent-free systems, and often work under mild temperatures.

Traditional catalysts often require harsher reaction conditions, such as high temperatures, toxic solvents, or elevated pressures.

#### 3.1.6. Reduced environmental and economic impact

Lower solvent usage, easier recovery, and reusability make magnetic nanocatalysts more eco-friendly and cost-effective.

Conventional catalysts typically generate more chemical waste, require frequent replacement, and demand higher energy and purification costs.

#### 3.1.7. Efficient in multicomponent and one–pot reactions

Magnetic nanocatalysts excel in GBB/GBBR reactions, A^3^-couplings, oxidative cyclizations, and other one-pot transformations essential for imidazo[1,2-*a*]pyridine synthesis.

Many conventional catalysts struggle with selectivity, by-product formation, or poor activation in complex multicomponent systems.

#### 3.1.8. Faster reaction rates and higher yields

Nanoscale dimensions enable rapid mass transfer, efficient adsorption, and accelerated bond formation, often yielding >90% conversions.

Bulk catalysts often exhibit slower kinetics and lower yields due to limited dispersion and poor substrate contact.

#### 3.1.9. Synergistic multicomponent functionality

Magnetic catalysts can combine acidic, basic, redox, or metal-catalyzed properties in a single nanostructure.

Conventional catalysts typically offer only one type of catalytic activity, reducing versatility.

#### 3.1.10. Lower catalyst loading

Due to high intrinsic activity, magnetic nanocatalysts require small amounts (1–10 mol%).

Conventional catalysts often require larger quantities, increasing cost and waste.

## 4. Future perspectives and outlook

Future developments in magnetic nanocatalysis for imidazo[1,2-*a*]pyridine synthesis are expected to focus on the design of more precisely engineered nanostructures, including single-atom catalysts, bimetallic systems, and advanced core–shell architectures; deeper mechanistic exploration through *in situ* spectroscopy and computational modeling to unravel active-site behavior and reaction pathways; broader implementation of green reaction media such as water, deep eutectic solvents, and solvent-free systems; integration of magnetic nanocatalysts into continuous-flow reactors for scalable and industrially viable synthesis, while addressing challenges such as large-scale preparation costs, separation efficiency in continuous operations, and long-term catalyst stability; creation of hybrid catalytic platforms incorporating biocatalysts, photocatalysts, electrocatalysts, or ionic liquids for enhanced multifunctionality; expansion of substrate scope toward renewable feedstocks and structurally complex precursors; adoption of ultrasound-, microwave-, and mechanochemistry-assisted protocols for faster and more energy-efficient transformations; comprehensive evaluation of catalyst toxicity, environmental impact, and long-term operational stability; application of artificial intelligence and machine learning tools for predictive catalyst design; and development of magnetic nanoreactors and confined catalytic environments capable of delivering improved selectivity and reactivity in heterocycle construction.

In summary, as demonstrated across numerous studies, magnetic nanocatalysts consistently deliver high yields, broad substrate tolerance, low metal leaching, and economic advantages, positioning them as superior alternatives to traditional catalysts. Collectively, the advancements discussed in this review highlight the growing significance of magnetic nanocatalysis in heterocyclic synthesis and underscore its substantial potential to guide the development of environmentally benign and industrially viable routes to valuable imidazo[1,2-*a*]pyridine frameworks Overall, magnetic nanocatalysts are poised to play a central role in next-generation green heterocycle synthesis, and their integration with machine learning, artificial intelligence, and high-throughput experimentation will accelerate discovery of optimized catalytic platforms.

## Conflicts of interest

The authors declare have no conflicts of interest to disclose.

## Data Availability

No additional data or separate datasets were generated or analyzed during this study. All information supporting the findings of this article is included within the article itself.
